# Long-Lived Charge Separation and Ionosolvatochromism of Methylene Blue

**DOI:** 10.1021/acsomega.6c01448

**Published:** 2026-05-20

**Authors:** Victor H. Toledo, Cedric R. Leão, Paula Homem-de-Mello, Otaciro R. Nascimento, Iseli L. Nantes

**Affiliations:** † Centro de Ciências Naturais e Humanas, Universidade Federal do ABC, Avenida dos Estados, 5001, Bloco A, Torre 3, Santo André, SP 09280-560, Brazil; ‡ Centro de Engenharias e Ciências Sociais Aplicadas, Avenida dos Estados, 5001, Bloco A, Torre 1, Santo André, SP 09280-560, Brazil; § Instituto de Física de São CarlosUniversidade de São Paulo, Av. João Dagnone 1100, São Carlos, SP 13563-120, Brazil

## Abstract

Methylene Blue (MB^+^) is a thiazine dye that has gained significant interest due to its diverse applications in health and technology. Here, two important findings regarding the formation of MB^+^-derived species are presented: photoinduced long-lived charge separation in organic solvents and the mechanism of the dye’s conversion to a red form. Previous studies have relied on ultrafast techniques—such as flash photolysis and transient adduct formation—to detect MB^+^’s excited states and radical intermediates. We now report on the production of three MB^+^-derived species formed after the exchange of the counterion Cl^–^ to OH^–^ in aprotic solvents, DMSO and toluene: MB^2+•^, MB^•^, and methylene red (MR^+^). The long-lived free radicals generated by charge separation and stabilized by π*–π* interactions were detected by the steady-state UV–visible spectroscopy and direct continuous-wave electron paramagnetic resonance (CW-EPR) spectroscopy. MR^+^ is an ionosolvatochromic species formed by trimers and tetramers of [MB^+^]­[OH^–^] in DMSO and toluene. Theoretical calculations supported the spectral assignments of radical cations and aggregates. These findings demonstrate a novel approach to stabilizing MB^+^ radical cations and aggregates, and to the ionosolvatochromism of MB^+^, thereby unlocking new avenues for its application in catalysis, synthetic chemistry, postlithium batteries, and biomedical technologies.

## Graphical abstract

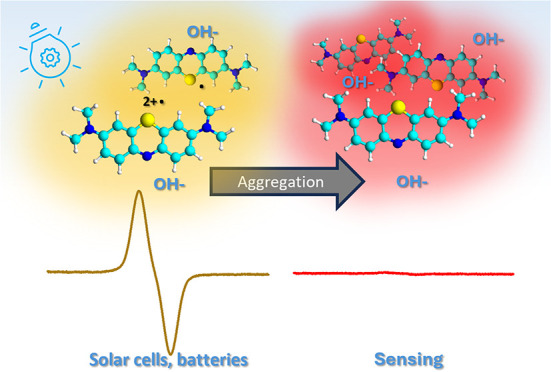

## Introduction

Methylene Blue (3,7-bis­(dimethylamino)-phenothiazin-5-ium chloride), represented as MB^+^, is a heterocyclic aromatic compound that has gained scientific interest since its synthesis in the late 19th century. The MB^+^ ability to bind to biomolecules and biological structures^1,2^ and to generate singlet molecular oxygen enables its use as a sensitizer in photodynamic therapy for cancer^3–5^ and in infectious diseases.^4^ MB^+^ is also used as a staining agent in cytology and histology. Medical and technological applications of MB^+^ include sensing,^6–9^ imaging,^10^ diagnosis,^11^ and solar cells.^12,13^ The photochemical and photophysical properties of the dyes are modulated by their aggregation states.^9,14–16^ In addition, its role as a redox indicator has enabled researchers to investigate electron-transfer mechanisms and reaction kinetics.^17,18^ Furthermore, MB^+^ applications as a model of dye pollutant in environmental science underscore this dye’s versatility and relevance in addressing global health and ecological challenges.^19,20^

The growing interest in the photoinduced redox reactions and solvatochromism of MB^+^ in technological research stems from the exceptional responsiveness of its dye to light and solvent environments, which enables precise control over its electronic and optical properties. The ability of MB^+^ and other dyes to undergo reversible redox transformations upon light exposure makes them promising candidates for applications in photocatalysis, solar energy conversion, and smart sensors, where dynamic electron transfer is essential.^21–23^ Simultaneously, its solvatochromic behavior—manifested as shifts in absorption spectra depending on solvent polarity—offers a powerful mechanism for designing responsive materials and molecular probes that adapt to environmental changes.^24^ These features have catalyzed innovations across fields such as optoelectronics, nanotechnology, and biomedical engineering, where MB+’s tunable behavior under varying conditions supports the development of advanced functional devices, including light-activated drug-delivery systems, solvent-sensitive coatings, and real-time diagnostic platforms.

The photophysical and photochemical properties of MB^+^, as well as those of other phenothiazinic dyes, are modulated by molecular aggregation states.^9,14–16^ Recently, it was demonstrated that the photoconversion of MB^+^ loaded into Chelex 100 beds to its two-electron-reduced leucoform, LMB. The formation of LMB involved a type I mechanism, in which the stabilization of the radical cation (MB^2+•^) and one-electron reduced free radical (MB^+•^) in dye aggregates facilitated the formation of LMB. The formation of LMB was postulated to occur by MB^+•^ dismutation or the abstraction of a second electron.^25^ The system was demonstrated to function as a photochemical reactor for the chemical synthesis of H_2_O_2_ and D_2_O_2_, as well as for the formation of anisotropic iridescent gold nanoparticles (GNPs).

In the present study, the extraction of leuco-Methylene Blue (LMB) entrapped in Chelex 100 using DMSO and toluene revealed the stabilization of MB^2+•^ for hours, followed by dye ionosolvatochromism to a red form.

## Materials and Methods

### Chemicals

Methylene Blue (C_16_H_18_N_3_SCl.3H_2_O), Chelex 100 resin, Amplex red, sodium hydroxide (NaOH), hydrochloric acid (HCl), and deuterium oxide (D_2_O) were purchased from Sigma-Aldrich Corp. (St. Louis, MO, USA). Deionized water (Millipore mixed-bed ion exchanger) was used to prepare all of the aqueous suspensions and solutions. Measurements of pH were made using a combined glass electrode (Orion Glass pH SURE-FLOWTM), and OrionFilling Solutions were used to fill the reference electrode (ROSSTM, model 8102). The potentiometer was calibrated using METREPAK pHydrion standard buffer solutions (Brooklyn, NY, USA). Acetone and DMSO were obtained from Synth. DMF and toluene were obtained from Merck.

### Experimental Details

#### LMB Extracted from Chelex 100

The cationic dye Methylene Blue has a binding affinity for a styrene-divinylbenzene copolymer containing paired iminodiacetate ions (Chelex 100). Previously, it was determined that 0.32 mg of MB^+^ can be removed from an aqueous solution by 100 mg of the resin (MB^+^/Chelex mass ratio = 0.0032). Chelex 100 exchanges Na^+^ for MB^+^ present in aqueous solution. In a typical experiment, 500 mg of Chelex 100 was added to 5 mL of 1 mM MB^+^ aqueous solution. After shaking, the dye was removed from the water, and the resin turned blue. The irradiation of MB^+^/Chelex converted the dye to the leucoform, LMB. Subsequently, water was removed under a N_2_ atmosphere to prevent dye oxidation, and 5 mL of DMSO or toluene was added to the resin, which was then shaken for 5 min to extract the dye from Chelex 100.

#### Blue Bottle Experiment

One hundred seventy microliters of MB^+^ (3.1 mM in ethanol) was added to 10 mL of a 0.2 M glucose solution in 0.7 M NaOH. After the formation of LMB by MB^+^ reduction by glucose, 5 mL of toluene was added to the solution. The flask was shaken, and phase separation occurred after resting. The toluene and aqueous phases were analyzed by UV–visible spectroscopy.

#### MB^+^ Chloride/Hydroxide Exchange in DMSO

A 100 μM solution of MB^+^ in 5 mL of DMSO was prepared from a 1/10 dilution of a 1 mM solution prepared by the addition of 12.8 mg of MB^+^ to 40 mL of DMSO. This solution was analyzed by UV–visible spectroscopy. In the following, 5 μL of a 100 mM NaOH aqueous solution was added to the DMSO solution until the initial blue color was converted to violet.

#### UV–Visible Absorption Spectrometry

The samples were characterized by UV–Vis spectroscopy using an Evolution 220 spectrophotometer (Thermo Scientific) with a standard liquid analysis configuration, employing quartz cuvettes with optical path lengths of 1–10 mm. The analyses were carried out in the range of 190–1100 nm, with a spectral resolution of 1 nm and an integration time of 0.01 s.

#### Electron Paramagnetic Resonance (EPR) Spectrometry

Direct EPR measurements of MB+ free radicals were performed using an EPR Varian E-109 X-band system. The measurement conditions were a gain of 5 × 10^3^, a modulation amplitude of 0.01 mT, a microwave power of 5 mW, and a time constant of 0.064 ms, with a room-made digital detection system at room temperature. The EPR signals of PITCH and MB^+^ were corrected for gain and microwave power.

The structures of MB^+^ and its complex aggregates were simulated using Density Functional Theory (DFT) with hybrid range-separated potential wB97X-D3BJ with Grimme’s correction for dispersion interactions,^26^ as implemented in the Orca software.^27^ To describe atomic orbitals, we employed a triple-ζ localized basis set, def2-tzvp. The solution medium (DMSO) was emulated through the conductor-like polarizable continuum model CPCM-SMD. For the optical absorption analysis, we used Time-Dependent (TD-DFT) with 100 states.

## Results and Discussion

Beads of the cation-exchange polymer Chelex 100 completely adsorb the cationic dye MB^+^ from an aqueous solution that is converted to the leucoform (LMB) under irradiation with visible light.^25^ LMB adsorbed to Chelex 100 was extracted from the samples using DMSO and toluene as described in the Materials and Methods (Figure 1a,b, respectively).

**Figure 1:**
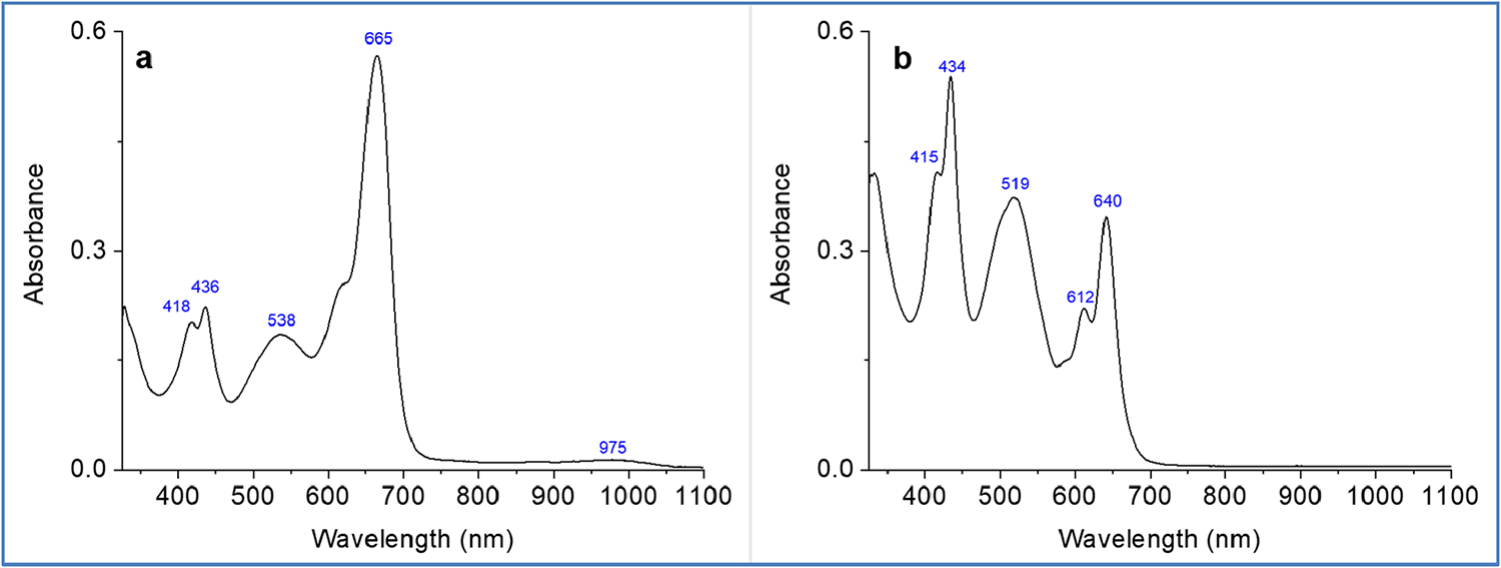
Spectra of MB^+^ after its extraction from Chelex 100. (a) In DMSO; (b) in toluene, both obtained immediately after extraction from the resin with conversion of MB^+^ to LMB.

The spectra presented in Figure 1a,b show bands at 418, 436, 665, and 975 nm for the dye in DMSO, and at 415, 434, 612, and 640 nm for the dye extracted with toluene. In the samples extracted with DMSO and toluene, broad bands with peaks at 538 and 520 nm, respectively, were also present. Samples of LMB and MB^+^ (maintained in the dark) entrapped in Chelex 100 were extracted with DMSO, and the respective spectra were obtained after 30 min, 5 days, and 12 days of dark rest (Figure 2a,b).

**Figure 2:**
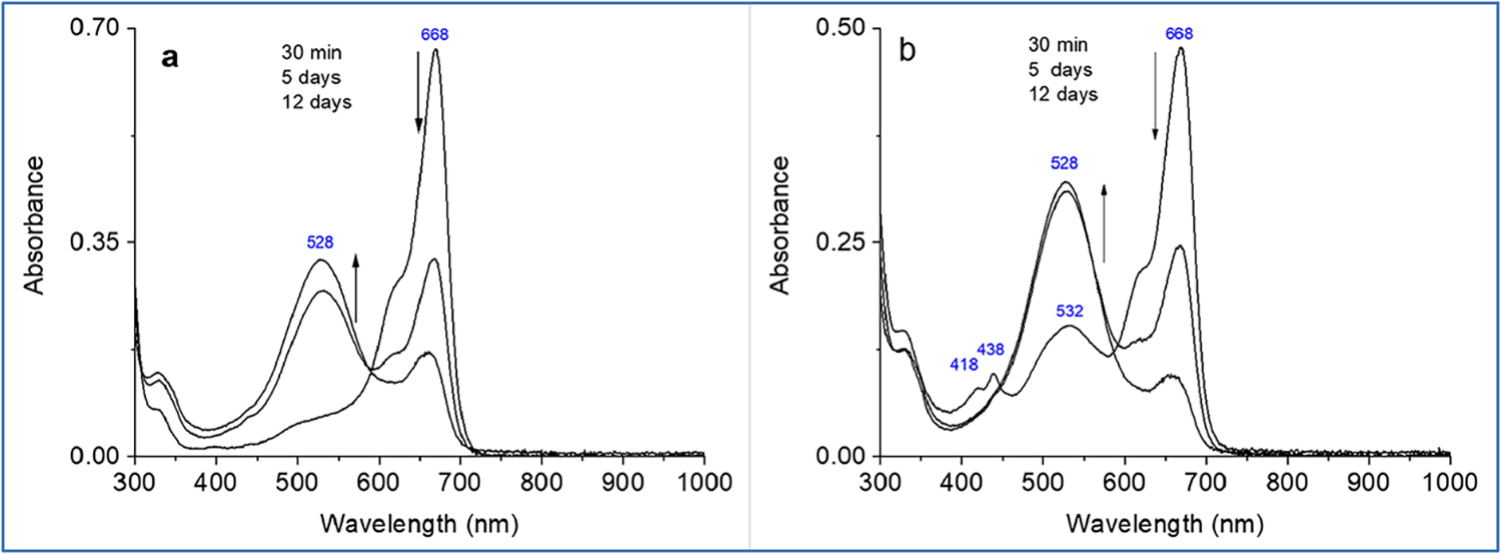
Time-dependent spectral changes of MB^+^ after its extraction from Chelex 100. (a) Without previous irradiation with visible light. (b) Extracted after conversion of MB^+^ to LMB by irradiation with visible light.

The spectrum of MB^+^ extracted from Chelex using DMSO and run after 30 min of resting does not show detectable bands at 400 and 900 nm (Figure 2a). The spectrum of LMB extracted from the Chelex-entrapped LMB obtained after 30 min of resting exhibits a band at 400 nm (Figure 2b) with an intensity 60% lower than that of the spectrum obtained immediately after extraction (Figure 1a). In contrast, the low-intensity band at 900 nm is no longer detectable (Figure 2b). These structured bands at 400 nm and the broad band at 900 nm are associated with the stable radical cation of phenothiazines.^28^ The bands in the 400–450 nm spectral range correspond to a π→SOMO transition localized on the thiazine ring. This transition is strongly influenced by the S–N heteroatom pair, which stabilizes the radical cation and can be accompanied by a lower-energy charge-transfer band (500–550 nm). The substituents on the phenyl rings influence the spectral peaks in the 400 nm range.^29^ The band at 900 nm is a fingerprint of the radical cation of aromatic compounds in the aggregated state stabilized by the π-stacking of heteroaromatic molecules.^30^ This low-intensity band became undetectable due to the significant decay of the radical cation over 30 min of resting. The visible absorption band of the MB^+^ at the spectral region of 600 nm with a shoulder at higher energy is assigned to a charge transfer (CT), partly forbidden n→π* transition, and n→π* transitions from amino groups.^31^

After 12 days of resting, the bands associated with MB^2+•^ disappeared, probably by charge recombination of MB^•^ and MB^2+•^. The bands in the 600 nm region, related to the MB^+^ monomer and dimers, decreased after 5 and 12 days of resting concomitant with the increase, narrowing, and blue shift of the band in the spectral region of 500 nm. After more than 12 days of rest, the MB^+^ sample in DMSO exhibited a spectrum with only a broad band in the 500 nm spectral region. This sample exhibited a red color (methylene red, MR^+^) similar to that described by Regmi et al. (2025).^24^ The MR^+^ band is broad, with a shoulder indicating spectral overlap from different species (Figure 3). After an equal time of resting, the MB^+^ sample extracted with toluene also exhibited a red color with a broad band peaking at 536 nm (Figure S1). The association of bands in the 500 nm spectral region with MB^+^-derived photoproducts was excluded by the restoration of the MB^+^ spectrum upon addition of water in a water-dependent manner (Figure S2). The water sensitivity of MR^+^ was also previously reported.^24^

**Figure 3:**
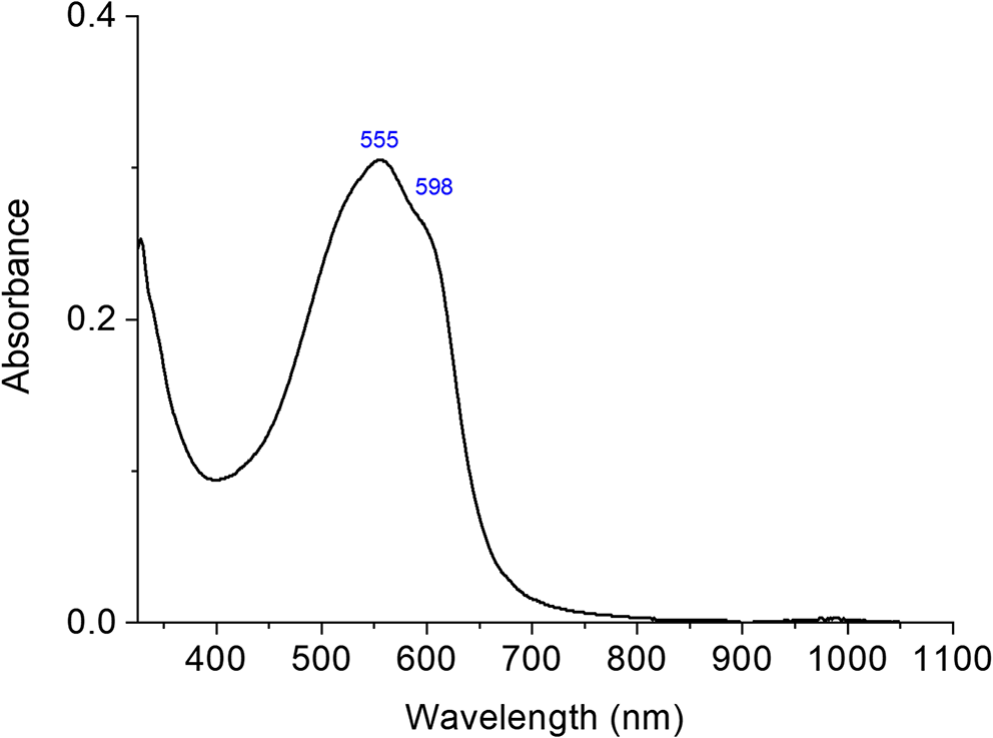
UV–visible spectrum of MR^+^ in DMSO stored for 30 days in the dark.

The reversion of MR^+^ to MB^+^ suggested that the bands in the 500 nm spectral region are associated with dye aggregates rather than with the formation of a photoproduct.

Considering that the broad band in the 500 nm spectral region could be associated with larger aggregates such as trimers and tetramers, the time-dependent increase in absorbance in this region was compared in DMSO, DMF, and acetone (Figure S3a–c). In this assay, MB^+^ in Chelex 100 was extracted with the cited solvents and analyzed by UV–visible spectroscopy immediately, after 4 and 10 days; all procedures were performed in the dark. The time-dependent conversion of MB^+^ monomers and dimers (blue colored) into the species absorbing in the spectral region of 500 nm (MR^+^) was favored in the following order: in DMSO > DMF > acetone (Figure S3a–c).

To corroborate the assignment of the bands in the spectral regions at 400 and 900 nm to the presence of free radicals, a sample obtained immediately after the LMB extraction was analyzed by CW-EPR (Figure 4a,b). The EPR spectra were simulated, revealing the presence of MB^•^ and MB^2+•^ signals. The lower percentage of MB^•^ relative to MB^2+•^ is consistent with partial reoxidation of MB^•^ via electron transfer to molecular oxygen and supports the assignment of the gradual disappearance of MB^2+•^ to charge recombination.

**Figure 4:**
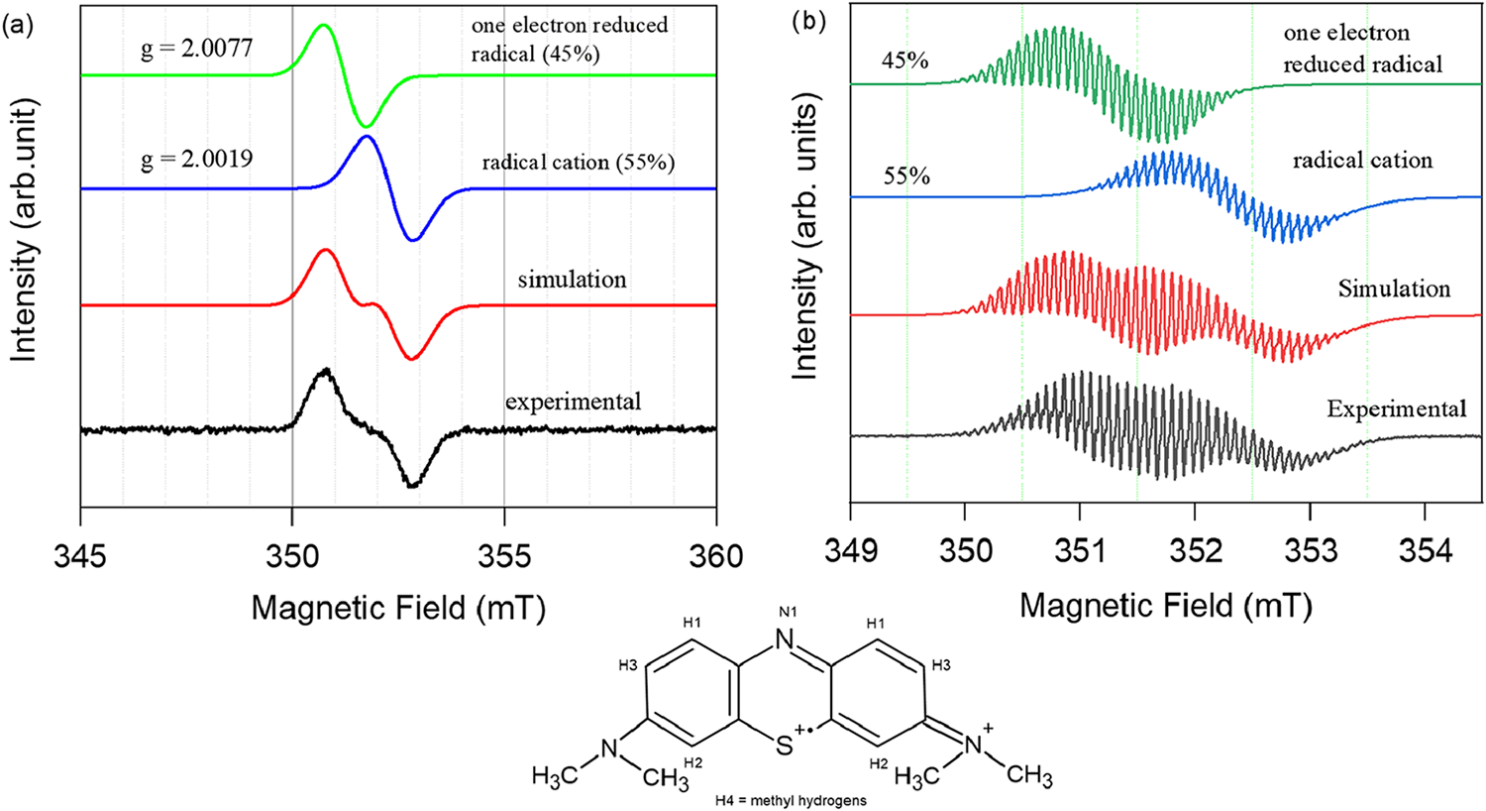
EPR spectrum of MB^+^-derived free radicals in DMSO. (a) EPR spectra measured at room temperature with overmodulation (AM = 0.5 mT). Simulation using derivative Gaussian line shape (Lw_anion_ = 0.998 mT, Lw_cation_ = 1.09 mT). Radical percentages are obtained by double integration of the simulated spectra. (b) EPR spectra measured at room temperature with a modulation (AM) of 0.025 mT. The simulation was performed using the EasySpin program. Radical percentages are obtained by double integration of the simulated spectra. The MB^2+•^ structure shows the identified nitrogen and equivalent hydrogen nuclei interacting with the unpaired electronic spin of the MB^+^-derived free radicals.

Figure 4a,b shows the EPR spectra of MB^+^-derived free radicals measured at modulation amplitudes of 0.5 and 0.025 mT, the simulated experimental data, and the contributions of MB^•^ and MB^2+^. The simulation parameters are shown in Table 1. The EPR spectra identified the unpaired electron in the sulfur atom and the interacting nitrogen and equivalent hydrogen nuclei. The characterization of sulfur-centered unpaired electron of MB^+^-derived free radicals was also corroborated by EPR using DMPO spin trapping (Figure S4a,b). The EPR parameters obtained from simulations of the immobilized MB^+^-DMPO adduct were in good agreement with the experimental spectrum and corroborated the assignment of a sulfur-centered free radical.^32^ The EPR signal obtained for the fresh solution (Figure S5a,b, violet lines) decreased drastically in the MR^+^ samples in DMSO and toluene (Figure S5a,b, red lines), indicating that the MR^+^ band does not have a contribution of MB^+^-derived free radicals.

**Table 1:** EPR Parameter Used to Simulate the MB^•^ and MB^2+•^ Spectra

parameter	g	Lw^a^	*A*_N1_(1)	*A*_N2_(2)	*A*_H1_(2)	*A*_H2_(2)	*A*_H3_(2)	*A*_H4_(12)
cation	2.0019	0.0196	0.515	0.160	0.084	0.084	0.082	0.079
anion	2.0077	0.0153	0.554	0.154	0.088	0.098	0.094	0.077

a Line width (Lw) and hyperfine constants (*A*_*ij*_) are in mT. Index *j* is shown in the molecular structure in Figure 4. The numbers in parentheses are the number of equivalent nuclei.

LMB was also obtained by the blue bottle experiment and extracted with toluene. In this condition, LMB is obtained by MB^+^ reduction with glucose, followed by the addition of toluene and shaking. In this condition, a phase separation occurred with MR^+^ concentrated in the toluene phase. Figure 5 shows the MR spectra in the toluene phase (black line) and the water phase (red line). The spectrum in the water phase is consistent with the products of glucose oxidation. The MR spectrum of toluene also shows bands assigned to MB^2+•^.

**Figure 5:**
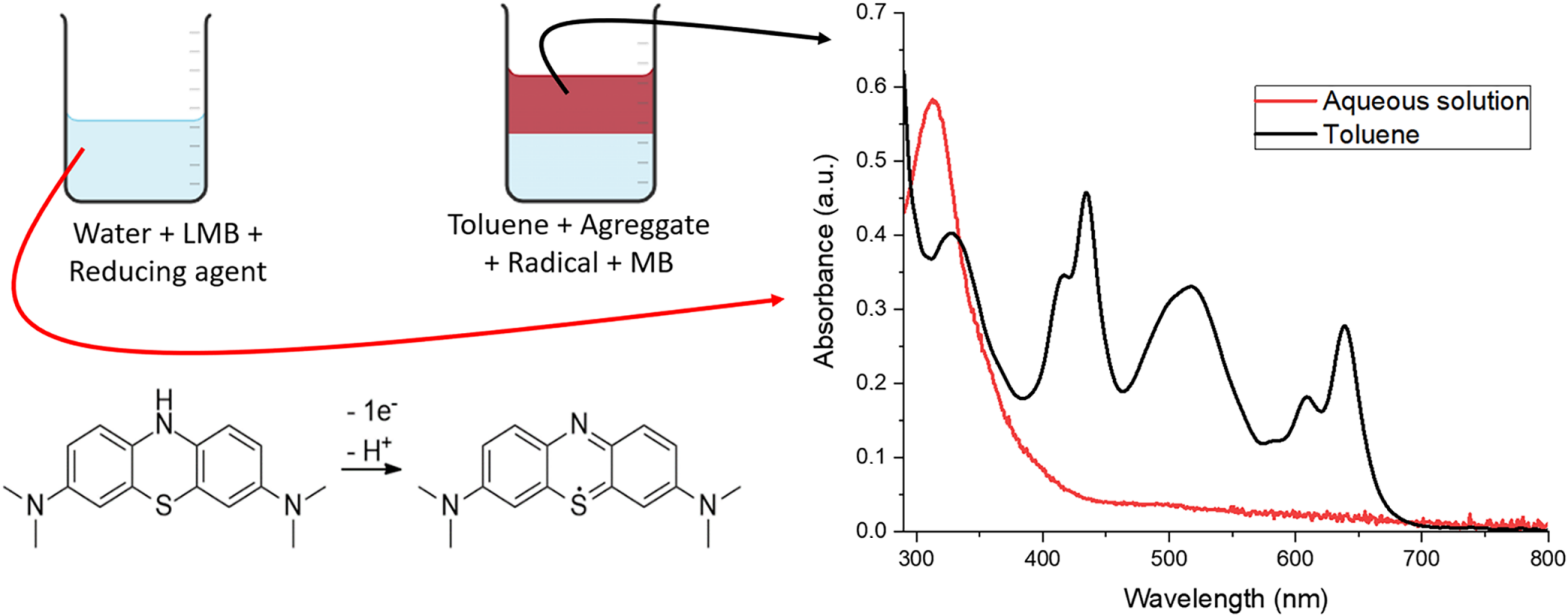
Spectra of MB^+^ extracted from an aqueous solution of LMB produced by the blue bottle experiment, with a schematic representation of the blue bottle experiment.

Considering that Regmi et al.^24^ obtained MR^+^ by exchanging the chloride counterion of MB^+^ with oleate and dissolving it in DMSO, it was important to conduct an additional experiment to investigate whether the MR^+^ obtained from LMB resulted from counterion exchange.

A solution of MB^+^ with chloride as the counterion was prepared in DMSO, and the spectrum is shown in Figure 6a (dotted black line). In DMSO, [MB^+^]­[Cl] exhibits a band peaking at 670 nm, consistent with the dye in a less polar solvent than water and predominantly in the monomeric form. Subsequently, 30 μL of 1 M NaOH was added to 5 mL of a 100 μM [MB^+^]­[Cl] solution in DMSO, resulting in the spectrum shown in Figure 6a (violet, dotted line). This spectrum exhibits bands at 420 and 440 nm, assigned to MB^2+•^, a band at 662 nm, and a band at 604 nm, attributed, respectively, to monomers and dimers of the dye, and a broad band peaking at 548 nm. Considering that free radical signals are not associated with MB^+^ species absorbing at the 500 nm region, this spectrum should be assigned to larger dye aggregates of methylthioninium hydroxide ([MB^+^]­[OH^–^]), which is MR^+^. The spectrum of the MB^+^-derived species obtained after NaOH addition does not match precisely with those shown in Figure 1a. This was expected as the addition of NaOH brings a content of water that can differ from that present in the sample extracted from Chelex. Also, Na^+^ is not present in the sample extracted from Chelex. The MB^+^ in DMSO is blue and changes to a violet color after NaOH addition (insets of Figure 6a). The violet color results from the contribution of MR^+^, which peaks at 548 nm, and residual MB^+^. The MB^+^-derived species obtained after NaOH addition, a mix of free radicals, MR, and MB^+^, was also water sensitive, as shown by the gradient of blue lines in Figure 6a. EPR confirmed the presence of MB^2+•^ and MB^•^ (Figure S6, violet line) in the sample of MB^+^ in DMSO after the addition of NaOH. The EPR signal decreased after 24 h of resting (Figure S6, red line). The spectral changes observed with increased water additions are attributed to the formation of reverse micelle-like structures, as illustrated in Figure 6b. The presence of MB^+^-derived free radicals was confirmed by EPR, while the MB^+^ monomers and dimers spectra are also well characterized. Regmi et al. reported solvatochromism of MB^+^ in DMSO when its chloride counterion was replaced with oleate.^24^

**Figure 6:**
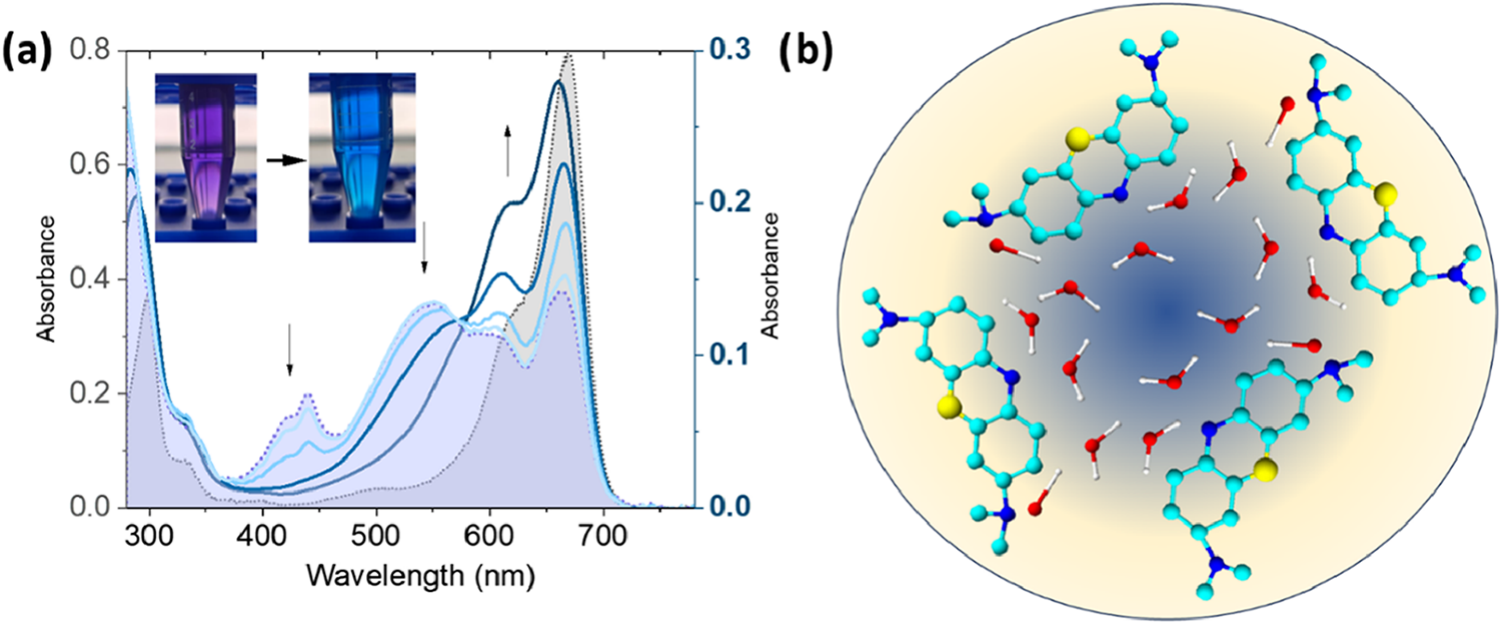
Spectra of MB^+^ in DMSO in different conditions. (a) [MB^+^]­[Cl^–^] in DMSO (black dotted line with area filled in gray), [MB^+^]­[OH^–^] in DMSO (violet dotted line with area filled in light violet), and the gradient of blue lines with progressive increase and decrease of bands after water additions indicated by the arrows. The inset shows the photos of the solution with zero and 5% of water. (b) Proposed reverse micelle-like organization of [MB^+^]­[OH^–^] in DMSO after water additions.

The authors did not describe the mechanism responsible for the MB^+^ solvatochromism observed exclusively for the oleate salt. In the present study, LMB extracted from the resin Chelex and in aqueous solution is expected to be devoid of chloride counterions, which could be replaced by OH^–^ via water deprotonation. Chelex 100 is an ion-exchange resin that binds the cationic dye by exchanging Na^+^ ions from its carboxylic groups with those of MB^+^. Thus, in Chelex 100, MB^+^ is associated with carboxylic groups via water molecules. The carboxylic groups of Chelex are covalently bound to the polymer chain, making it reasonable to assume that MB^+^ is extracted by organic solvents associated with OH^–^. MB^+^ with OH^–^ as the counterion, and possibly coordinated with a few water molecules, should form large aggregates, such as trimers and tetramers, in organic solvents such as DMSO and toluene, which are responsible for ionosolvatochromism.

Considering MR^+^ as aggregates (trimers and tetramers) of [MB^+^]­[OH^–^], the solvent-dependent stabilization of this species, not observed for [MB^+^]­[Cl^–^], is related to solvation, favoring ion-pairing and π–π stacking. MB^+^·OH^–^ aggregates in DMSO because OH^–^ is poorly solvated, forming tight ion pairs that reduce charge repulsion and promote π-stacking. MB^+^·Cl^–^ does not aggregate because Cl^–^ is strongly solvated by DMSO, leading to dissociated ions and stable monomeric MB^+^. Comparing the effect of solvents for [MB^+^]­[OH^–^] (Figure S3a-c) with the support of Table S1, toluene, the most apolar solvent used, strongly favors aggregation, as it cannot solvate ions and promotes π–π stacking. Aggregation decreases through DMSO and DMF as ion solvation improves. Acetone solvates both MB^+^ and OH^–^ well enough that monomers are favored, giving the weakest aggregation.

The spectral assignments of MB^+^-derived free radicals and aggregates, particularly larger ([MB^+^]­[OH^–^]) aggregates in DMSO as MR^+^ species, were investigated using DFT (Figure 7a–g). We performed several quantum-based atomistic simulations using density functional theory to evaluate the diversity of aggregates formed by MB^+^ monomers in the DMSO medium. The most representative initial configurations of MB^+^ aggregates are depicted in Figure 7a–g. After the atomic optimizations were conducted, we observed that structures (f) and (g) evolved to configurations very close to the structure (b), the antiparallel dimer. Therefore, we disregard them as metastable in DMSO. We also tested variants of the conformations (b), (c), and (d) with the monomers stacked in a mirror image, with the sulfur atoms on different monomers facing each other (not shown). We call these “parallel” structures, in opposition to the “antiparallel” ones, shown in Figure 7. The structure depicted in Figure 7b is 0.028 eV lower in formation energy than its parallel counterpart and 0.022 eV lower in energy than the “cross” dimer, indicated in Figure 7e. So, the “antiparallel” dimer is the most stable structure for dimers, as observed before for dimers extracted from crystals,^33^ and obtained in the gas phase and in water.^34^ However, because these energy differences are comparable to the average thermal energy at room temperature, we expect all of these structures to be present under experimental conditions.

**Figure 7:**
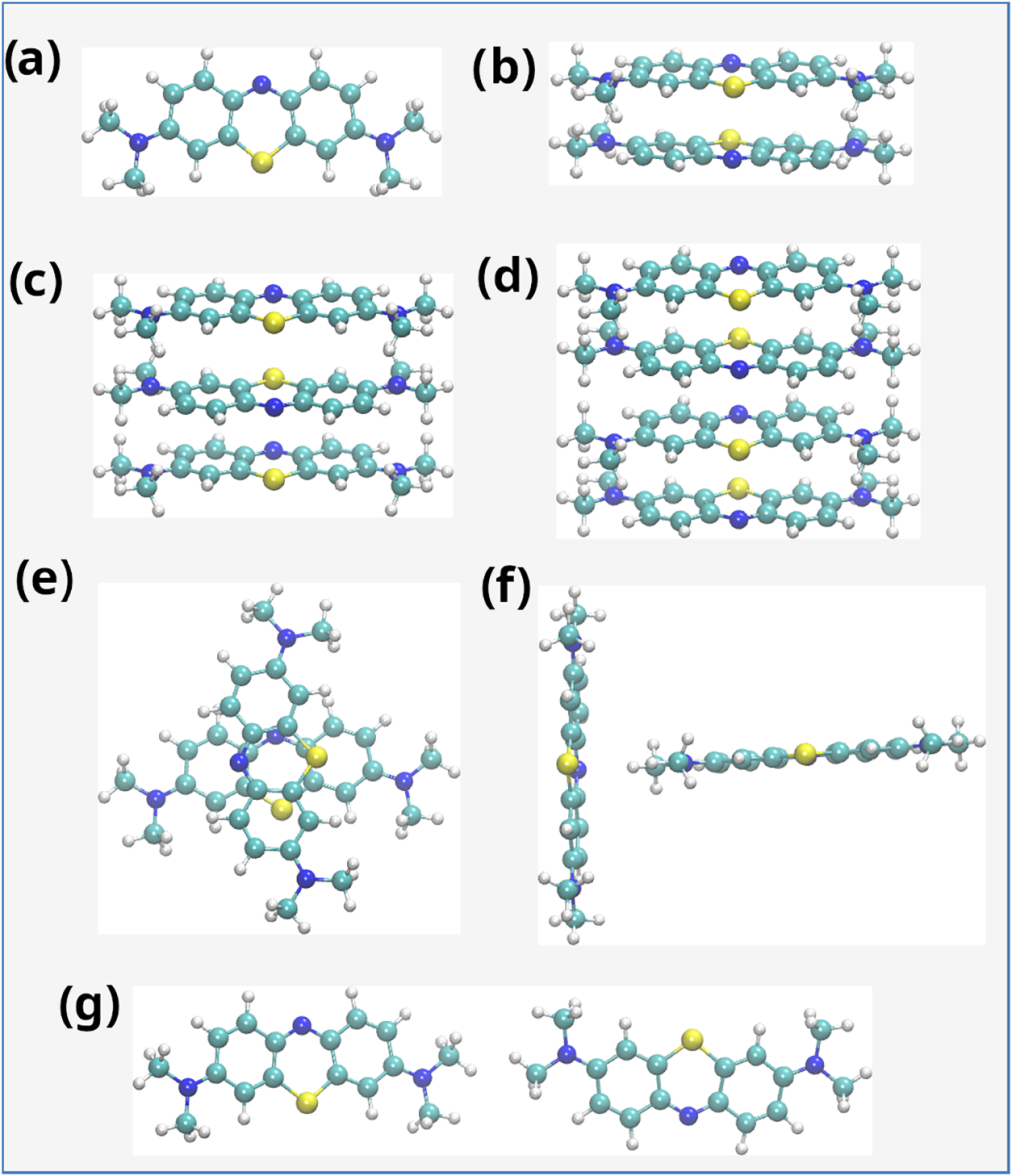
Initial configurations of the most representative MB^+^ aggregates were simulated. (a) Monomer, (b) H-dimer (inverted), (c) Trimer (antiparallel), (d) Tetramer, (e) ″cross″ dimer, (f) T-dimer, (g) J-dimer.

Figure 8 shows the binding energies per monomer for the stable, optimized structures (b), (c), and (d) in the antiparallel and parallel conformations. These binding energies are computed as

1 $$E_{B}=\frac{\left(E_{ag}-n\times E_{mon}\right)}{n}$$

where *E*_B_ is the binding energy per monomer, *E*_ag_ is the total energy of the aggregate in consideration, *E*_mon_ is the total energy of the monomer, and *n* is the number of monomers that compose the aggregate. As expected, and as shown in eq 1, the more negative the energy, the stronger the aggregate binding.

**Figure 8:**
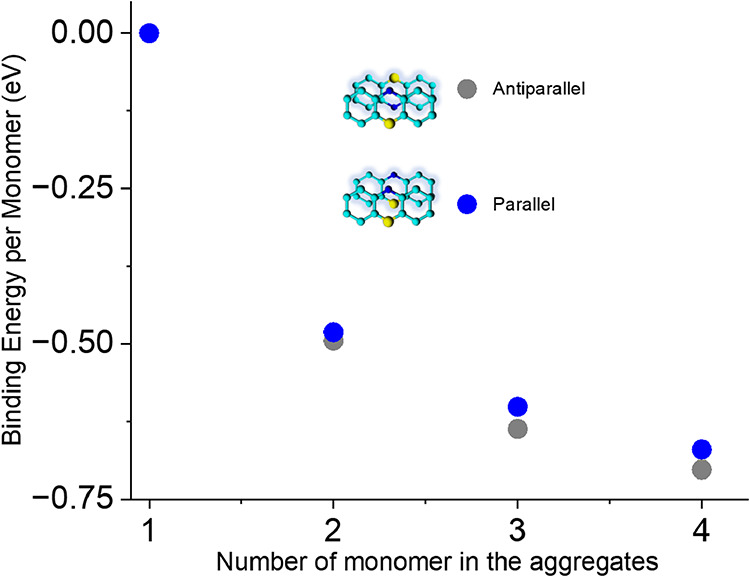
Number of MB^+^ monomers in the aggregates as a function of binding energy.

These results show that complexes of two, three, and four MB^+^ monomers, stacked as in H-aggregates, are highly stable in DMSO. Extrapolating the trendline shown in Figure 8, we infer that increasing the number of monomers decreases energy. Because the number of monomers does not scale linearly with function, the energy gain should eventually plateau and likely reverse for larger aggregates. TD-DFT calculations were used to simulate the optical absorption spectra of MB^+^ and its aggregates in DMSO. The MB^2+•^ and MB^•^ charged species were also simulated to support the experimentally observed absorption bands shown in Figures 1 and 6. Figure 9 shows the simulated absorption peaks with the largest oscillator strengths aligned with the absorption spectrum of MB^+^ extracted from Chelex with toluene. The simulated spectrum shows peaks that match the relative absorption energy of the MB species observed in DMSO and toluene.

**Figure 9:**
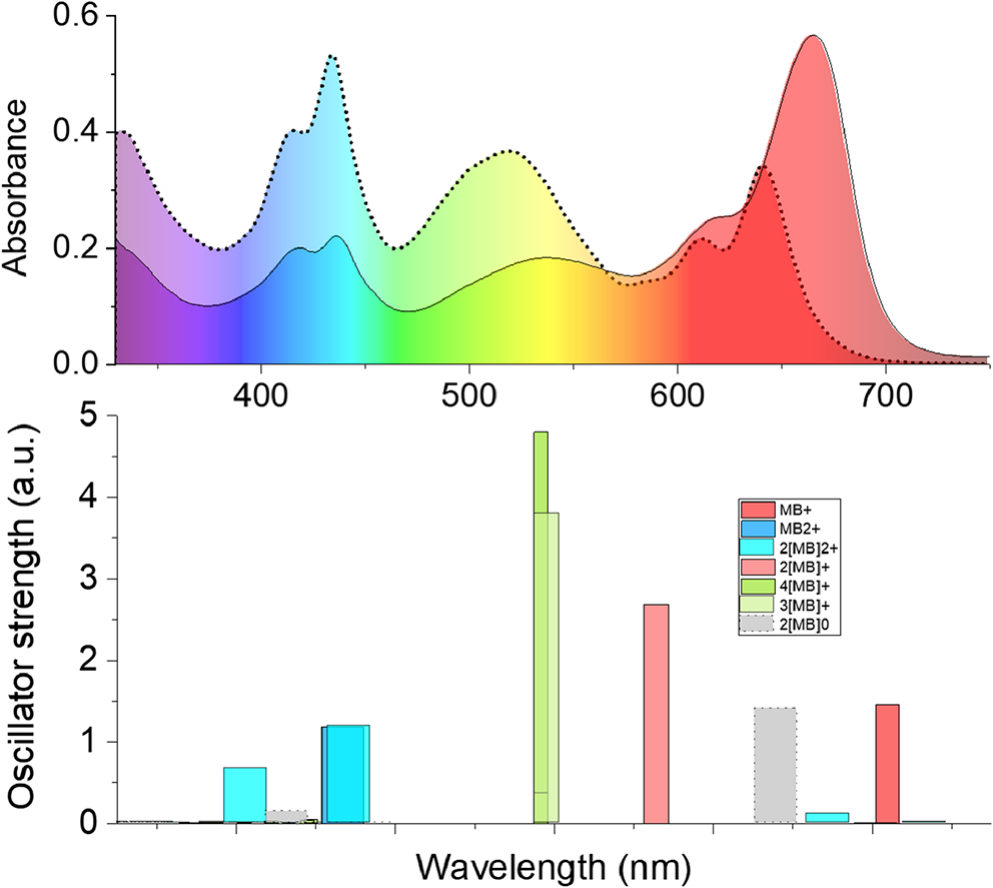
Absorption peaks obtained by TD-DFT simulations, associated with the MB^+^ species monomer in DMSO (black line) and toluene (dotted line), as well as its most stable aggregates. Peaks associated with free radicals of the monomer and the dimer are also shown.

Together with the EPR spectra, the DFT calculations support the assignment of the bands within the 400 nm region to MB^2+•^ and indicate that these bands can result from the contribution of monomers (MB^2+•^) and dimers [MB^2+•^]_2_. The simulated spectrum of the radical cation species lies between 400 and 415 nm. In Figure 1a, we observe peaks at 416 and 436 nm in the MB^+^ solution in DMSO, and at 415 and 434 nm in the toluene solution after removal from Chelex 100. It is important to emphasize that the absolute peak positions in simulations and experiments should not be directly compared. The relative ordering of the peaks and the comparative oscillator strengths are reliable.

Although MB^•^ has been identified in the direct EPR signal of the dye extracted from Chelex, from water in the blue bottle assay, after the addition of NaOH in DMSO solution, and as DMPO adduct, there was no absorption band evident to be assigned to that species. The DFT calculations demonstrated that the MB^•^ spectral contributions are overlapped in the spectral region of MB+ and [MB+]_2_ and MB^2+•^ and [MB^2+•^]_2_. We believe that, under experimental conditions, these species, MB^•^ and [MB^•^]_2,_ along with the MB^2+•^ and [MB^2+•^]_2_ counterparts, may be dynamically formed by splitting higher-order aggregates and regrouping; therefore, these EPR-active charged states should contribute to the absorption spectrum. Density plots obtained through DFT of the states involved in the optical transitions of the higher aggregates (Trimer and Tetramer), shown in Figures 10 and 11, as well as the plots of the optical transitions of the radical dimer, included in the Supporting Information (Figures S7 to S10), corroborate this hypothesis.

**Figure 10:**
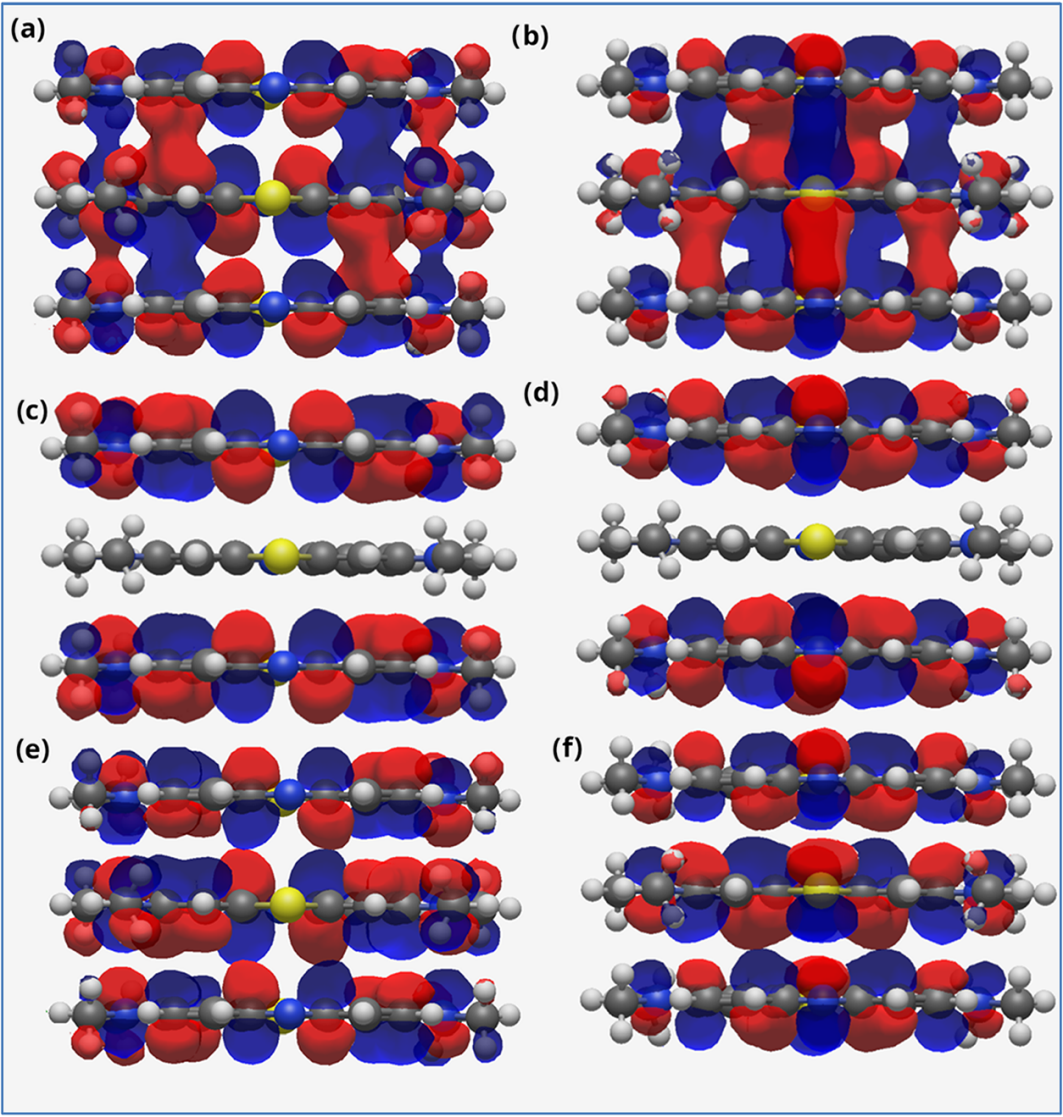
Plot densities of the transitions that make up the absorption peak related to the trimer. (a) Homo-3 to (b) LUMO; (c) HOMO–2 to (d) LUMO+2, and (e) HOMO to (f) LUMO+3.

**Figure 11:**
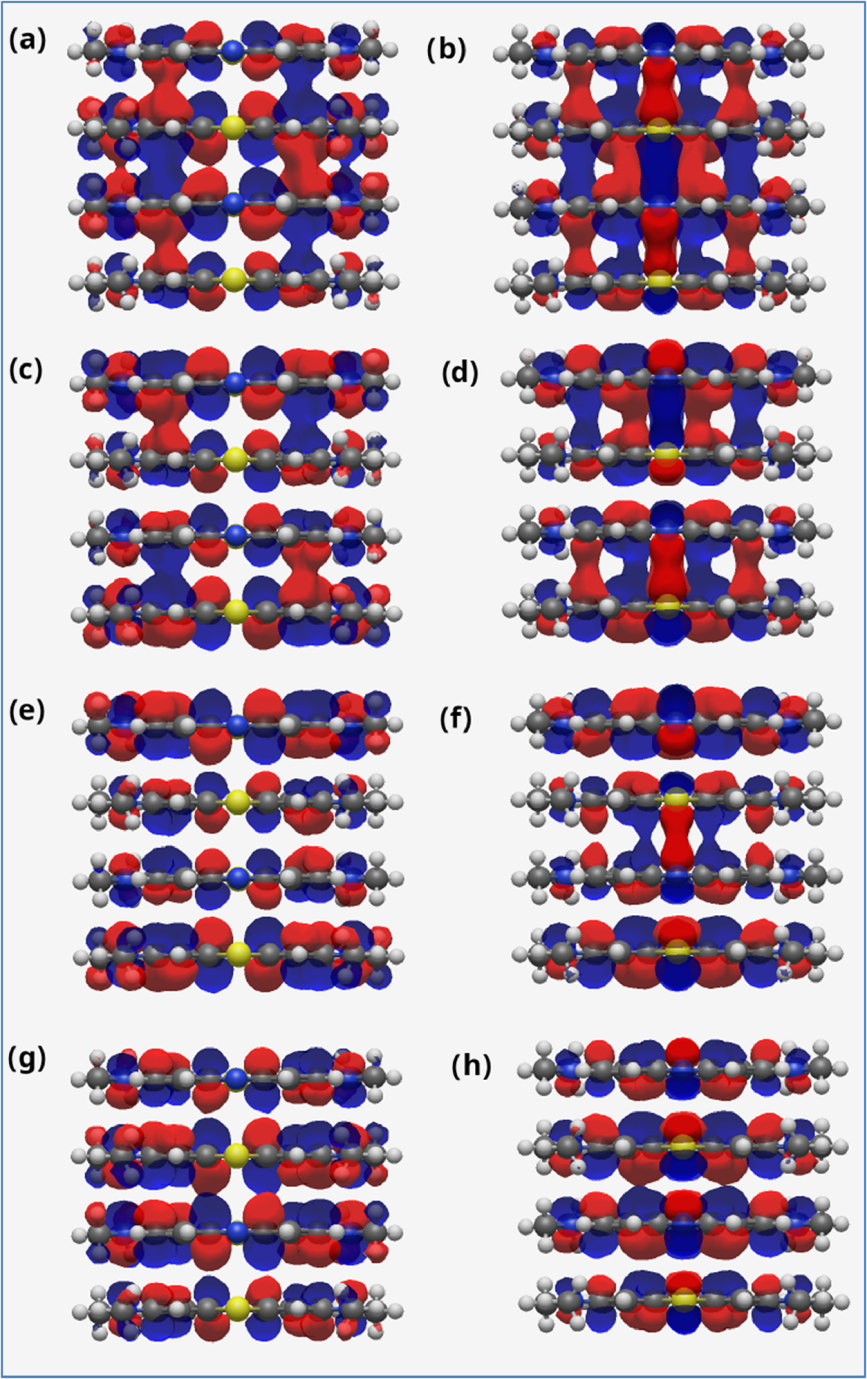
Plot densities of the transitions that make up the absorption peak related to the tetramer in Figure 10. (a) Homo-3 to (b) LUMO; (c) HOMO–2 to (d) LUMO+1, (e) HOMO–1 to (f) LUMO+2, (g) HOMO to (h) LUMO+3.

Regarding the broad band within the 500 nm region that increases during the resting of the dye extracted from Chelex by DMSO and toluene (Figures 2 and 4), DFT simulations corroborated the assignment of this band to spectral contributions of larger aggregates, trimers and tetramers, in this case ([MB^+^]­[OH^–^])_3_ and ([MB^+^]­[OH^–^])_4_ as evidenced by NaOH addition in MB^+^ solution in DMSO. The broad and featureless nature of this band is itself consistent with a distribution of aggregate sizes and/or geometries, where contributions from charge-transfer-mixed exciton states and geometrically disordered packing arrangements, such as oblique stacking, could also contribute to inhomogeneous broadening.^35,36^ While the current data do not allow definitive exclusion of such alternatives, the overall spectroscopic behavior, including the blue-shifted character of the band relative to the monomer and dimer absorptions, its progressive growth during extended rest, its solvent-dependent formation, and its reversibility upon water addition, is most parsimoniously interpreted as arising from higher-order H-type aggregation of [MB^+^]­[OH^–^], as supported by the TD-DFT calculations.

This breaking of aggregates into smaller components can actually be induced by optical absorption. Figure 10 shows the density plots of the states involved in the most relevant transitions of the trimer, as indicated in Figure 9. In Figure 11, we present plots of the border orbitals of the states involved in the transitions within the tetramer. Optical excitation induces a charge rearrangement that can depopulate bonding-state levels across layers and populate antibonding states, thereby weakening interlayer binding and leading to scission. The charged fragments that result from these aggregates can eventually reaggregate due to electrostatic interaction. In the Supporting Information, we present density plots of the other species whose absorption peaks are indicated in Figure 9 (Figures S7–S10).

Considering the stability of the aggregates, Figure 8 shows that tetramers and trimers are highly stable in DMSO, as these species were not converted to monomers or dimers after 10X dilution. Otherwise, density plots of the optical states involved in the transitions of MB^2+•^ and MB^•^ indicated that these free radical species should recombine to MB^+^ and [MB^+^]_2_ after dilution.

The photogeneration of LMB in Chelex results from a type I mechanism, in which a photoinduced charge separation occurs within the aggregates of MB^+^.^25^ In this mechanism, MB^2+•^ and MB^•^ are formed, and LMB results from MB^•^ disproportionation. Therefore, the extraction of LMB by DMSO or toluene in an air atmosphere could support the formation of MB^+^ and MB^•^ to total and partial LMB oxidation by O_2_ with subsequent formation of superoxide ion (O_2_^•–^) and hydrogen peroxide (H_2_O_2_). Considering the improbable extraction of MB^2+•^ from Chelex by low-polarity solvents, the formation of MB^2+•^ could result from the MB^+^ oxidation by unprotonated H_2_O_2_. Oxidation of MB^+^ by H_2_O_2_ was previously reported by Katafias et al.^37^ In this condition, MB^•^ could be formed by one-electron oxidation of LMB by O_2_, and MB^2+•^ could be formed by MB^+^ oxidation by HO_2_^–^. Consistently, significant free radical bands were not present in the samples resulting from the extraction of Chelex-entrapped MB^+^ in the dark, a condition in which hydrogen peroxide could not be formed. Although the above mechanism should not be disregarded, it does not support MB^2+•^ detection upon the addition of NaOH to the MB^+^ solution in DMSO. Therefore, the mechanism that can explain the free radical detection in all three assays described here is the total oxidation of water-coordinated LMB extracted from Chelex and from water (blue bottle assay) and the formation of [MB^+^]­[OH^–^] via water deprotonation. In the organic solvents, [MB^+^]­[OH^–^] species form aggregates, such as dimers, trimers, and tetramers, in which even room light can promote charge separation leading to the production of MB^2+•^ and MB^•^. For the first time, a condition in which long-lived MB^+^-derived free radicals are formed and could be detected by steady-state UV–visible spectroscopy and continuous-wave EPR spectroscopy. The charge recombination of MB^2+•^ and MB^•^ restores [MB^+^]­[OH^–^] in the aggregates, while monomers and dimers of [MB^+^]­[OH^–^] associate with these aggregates, leading to MR^+^ appearance (aggregates of [MB^+^]­[OH^–^]) to be the only spectral species detected after resting. When OH^–^ replaces chloride after the addition of NaOH in a DMSO solution of MB^+^, the aggregates are also formed, leading to charge separation in part of the [MB^+^]­[OH^–^] molecules. The present findings allow us to revisit the previous interpretation of Chelex-entrapped LMB conversion to a red species upon UV irradiation in an O_2_-depleted atmosphere.^25^ Previously, the UV-generated red species was attributed to the partial oxidation of LMB, specifically to MB^•^. According to XANES and NRM results, the dye is H- and J-aggregated in the Chelex matrix and is coordinated by water, linking it to the carboxylic groups of the resin. Under these conditions, the reoxidation of LMB aggregates restores the dye’s positive charge, thereby favoring water deprotonation and the formation of [MB^+^]­[OH^–^] aggregates entrapped in the resin. In Chelex, MB^+^ could coordinate with the carboxylic groups of Chelex, a condition also favorable to ionochromism. The proposed mechanisms are depicted in Scheme 1.

**Scheme 1:**
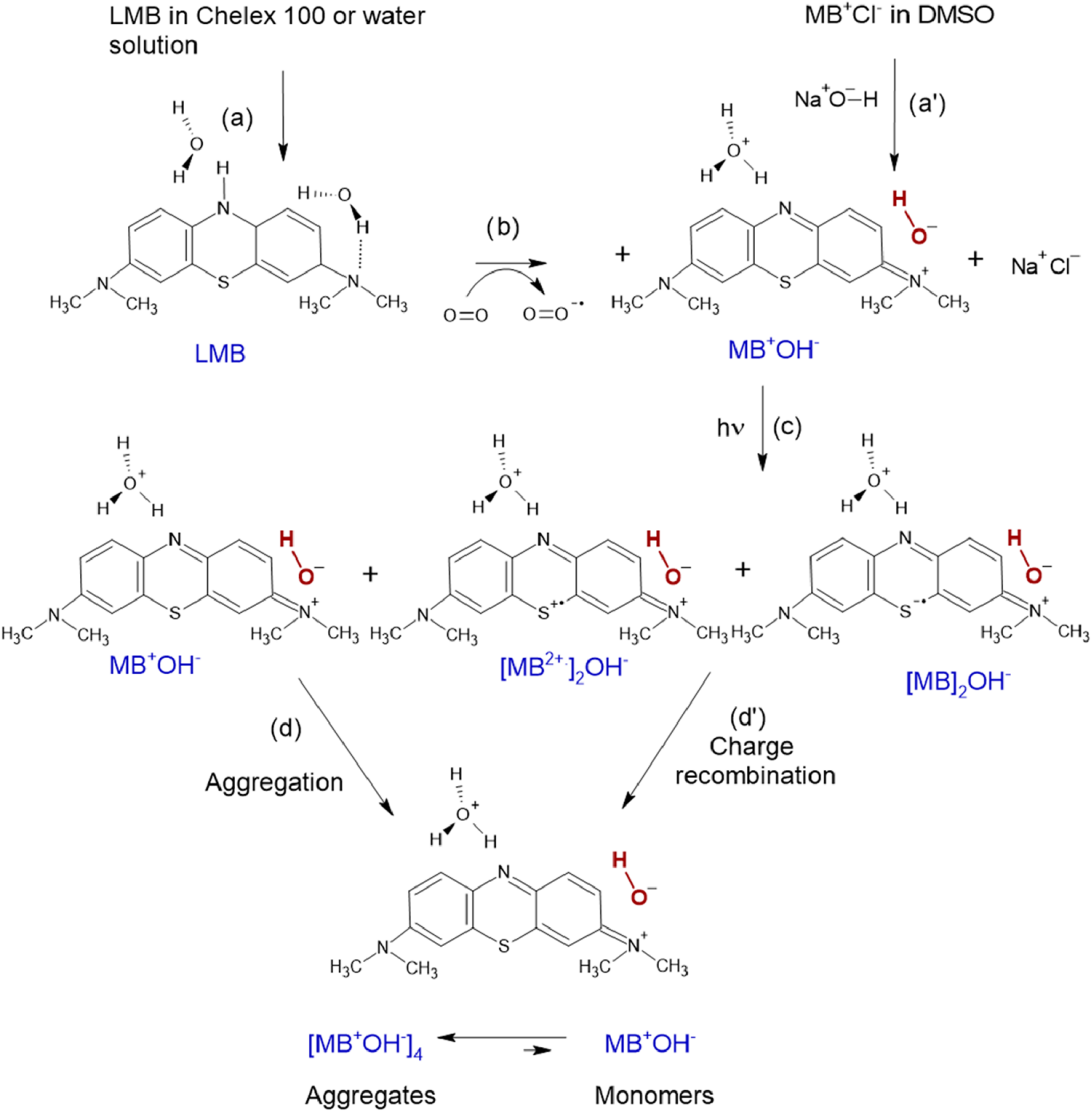
Schematic Representation of the Mechanisms of MR^+^ Formation Described in the Present Study^a^

a (a) Hydrated LMB hydrogen bonded with water in Chelex; (a′) MB^+^Cl^–^/Na^+^OH^–^ counterion exchange in DMSO by addition of NaOH to form MB^+^OH^–^ (MR); (b) oxidation of LMB by O_2_ during the dye extraction from Chelex by DMSO to form MB^+^OH^–^ (MR^+^); (c) light absorption of MB^+^OH^–^ aggregates leading to charge separation in the oxidized and reduced free radical species absorbing in the 400–450 nm range; (d) and (d′) respective increased concentration of MR^+^ aggregates monomers aggregation and charge recombination of oxidized and reduced free radical in aggregates. Only the water molecule involved in protonation was represented.

## Conclusion

The replacement of [MB^+^]­[Cl^–^] by an ROH^–^ counterion, as the hydroxide anion (OH^–^), yields the [MB^+^]­[ROH^–^] aggregates, which exhibit ionosolvatochromism toward MR^+^ and reversibly shift to the blue species upon water addition. In the present study, it was demonstrated that [MB^+^]­[OH^–^] can be formed by different assays: LMB extraction from Chelex polymeric matrix and from water, and by the addition of NaOH in a DMSO solution of [MB^+^]­[Cl^–^]. Additionally, MR^+^ can be formed in Chelex upon the reoxidation of LMB by UV irradiation. In DMSO, after NaOH addition, the low-polarity solvent enhances the interaction between Na^+^ and Cl^–^ ions, thereby facilitating the formation of [MB^+^]­[OH^–^]. It was also demonstrated that, in DMSO, toluene, and even in DMF and acetone, [MB^+^]­[OH^–^] forms trimers and tetramers, as shown by DFT calculations. During rest, trimers and tetramers become the dominant species due to charge recombination of MB^2+•^ and MB^•^, and to the gradual incorporation of [MB^+^]­[OH^–^] monomers and dimers into larger aggregates. This explains the broad band in the 500 nm spectral region, which results from the overlapping contributions of different [MB^+^]­[OH^–^] aggregates in varying proportions. Water molecules likely coordinate MR^+^ in DMSO and toluene. Still, the addition of more water to this system converts MR^+^ to a blue-colored species, probably by reorganizing the dye molecules from H-aggregates into a reverse micelle-like structure.

Given the multiple applications of MB^+^ in therapeutics and across various technologies, the present study is highly relevant for elucidating conditions that promote charge separation and stabilize the dye’s free radicals in a low-polarity microenvironment. The lipid bilayer of cell membranes may facilitate the formation of [MB^+^]­[OH^–^] species during dye incorporation, thereby promoting MB^+^-derived free radicals that contribute to cell death in PDT. For technological applications, the long-lived charge separation of MB^+^ has potential for applications in energy. Water sensitivity has previously been identified as a potential application of MR^+^ in sensing; however, the mechanism of MR^+^ formation was elucidated only in the present study. The possibility of promoting MB^+^ conversion to MR^+^ across different media, including solid matrices, opens diverse technological applications. A blue-shifted MB^+^ (MR^+^) absorbing at ∼530 nm could be used as a tunable chromophore in optical oxygen-sensing films or fibers, where the dye’s photophysics could be applied to detect oxygen concentration through fluorescence quenching, absorbance modulation, and photoredox cycling.

## Supplementary Material



## Notes

Additional UV–visible and EPR spectra; and DFT results (PDF)
