# Supplementary material for: Long-Lived Charge Separation and Ionosolvatochromism of Methylene Blue
Source: ACS Omega. 2026 May 20;11(22):32666–77. doi: 10.1021/acsomega.6c01448 (PMC13261577; doi:10.1021/acsomega.6c01448)
Supplement: Supplementary file 1 [file ao6c01448_si_001.pdf]

Supplemental Material

Long-lived Charge Separation and Ionosolvatochromism of Methylene Blue.

Victor H. Toledo[1], Cedric R. Leão[2], Paula Homem-de-Mello[1], Otaciro R. Nascimento[3] and Iseli L. Nantes*[1]

1. V. H. Toledo, P. Homem-de-Melo, I. L. Nantes
Centro de Ciências Naturais e Humanas
Universidade Federal do ABC.

Avenida dos Estados, 5001, Santo André-SP, Bloco A, Torre 3, 09280-560, Brazil.
E-mail: victorhlatoledo@gmail.com, paula.mello@ufabc.edu.br, *ilnantes@gmail.com and *ilnantes@ufabc.edu.br

2. C. R. Leão
Centro de Engenharias e Ciências Sociais Aplicadas.
Avenida dos Estados, 5001, Santo André-SP, Bloco A, Torre 1, 09280-560, Brazil.

E-mail: cedric.rocha@ufabc.edu.br

3. O. R. Nascimento
Instituto de Física de São Carlos
Universidade de São Paulo.
Av. João Dagnone 1100, São Carlos, SP, 13563-120.

E-mail: otaciro7f@gmail.com

Supplemental Results

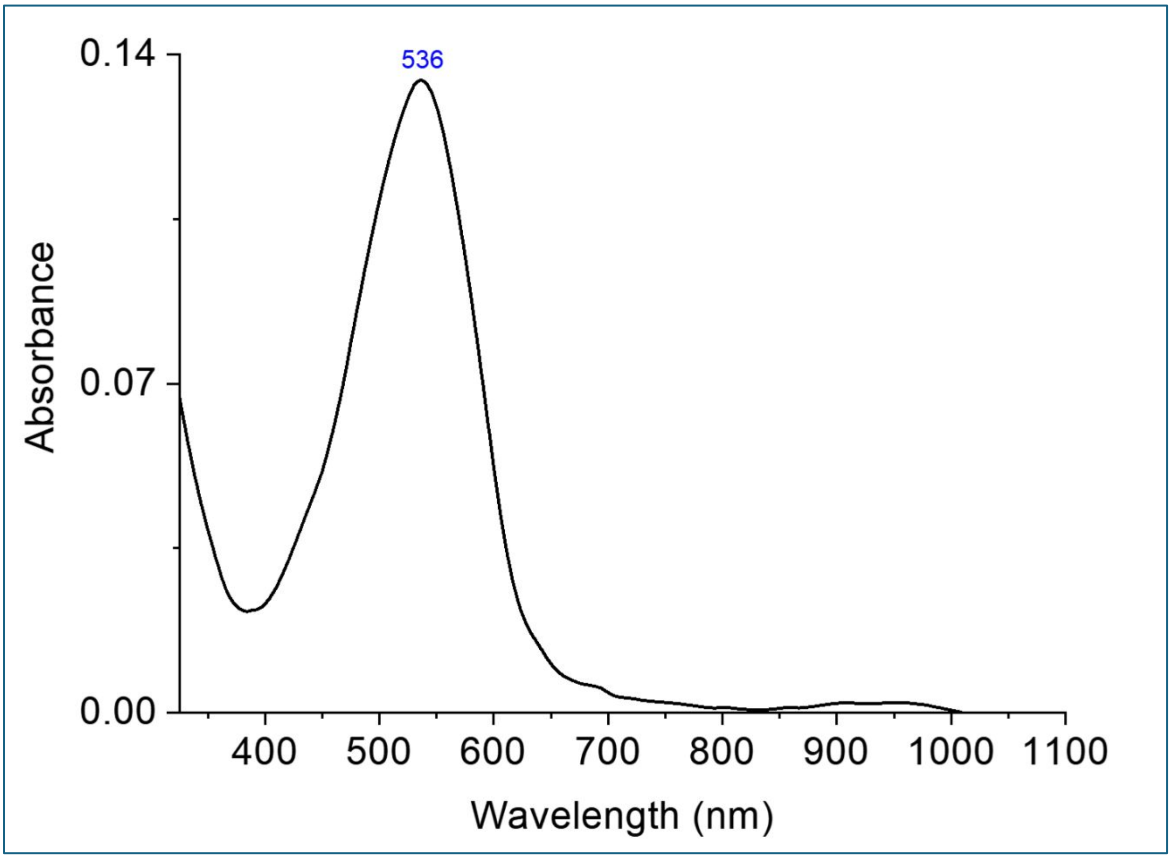

Figure S1. Spectrum of MR^+ in toluene resulting from storage for 12 days of LMB extracted from Chelex 100 using toluene.

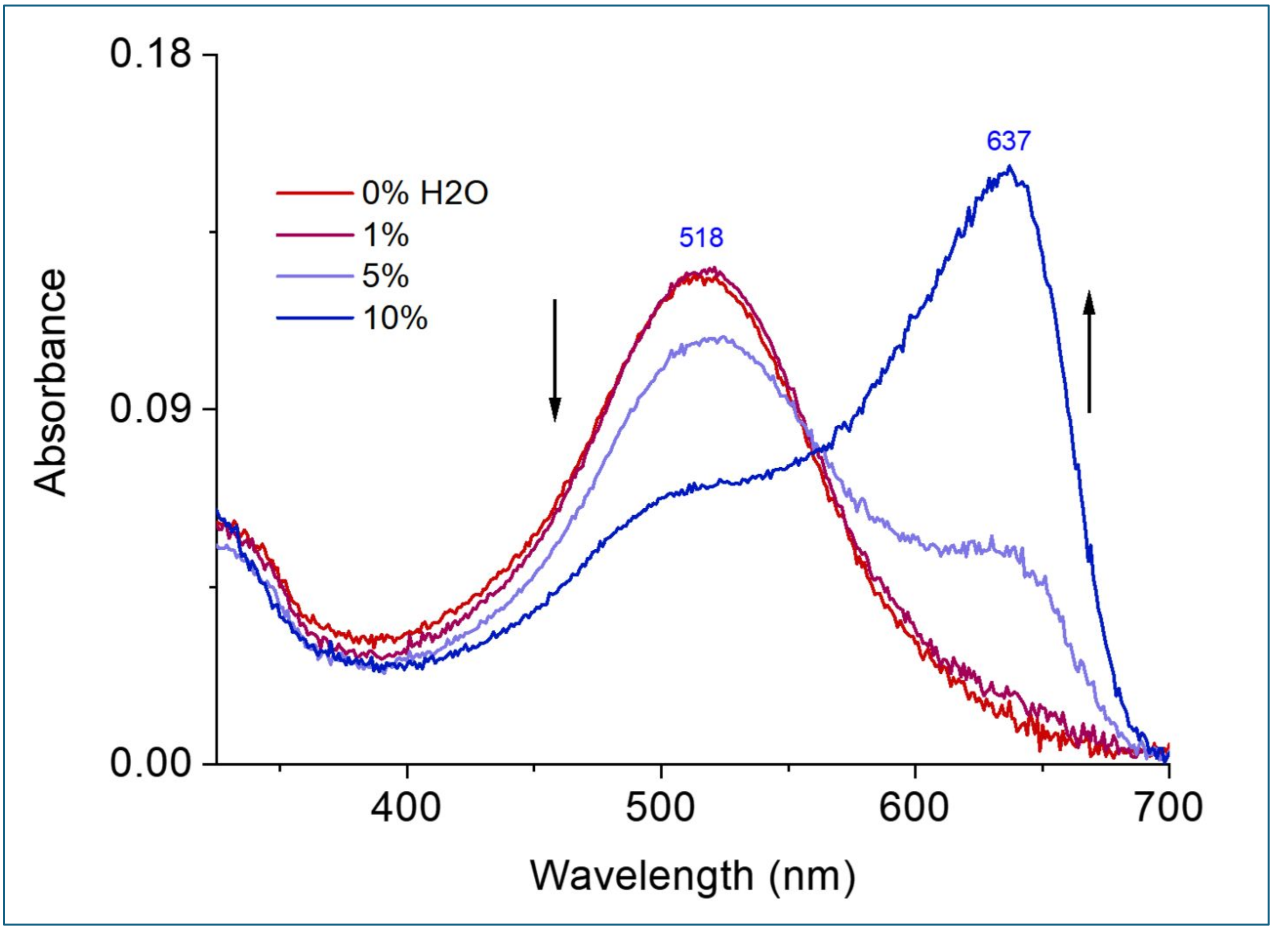

Figure S2. Spectral changes of MR^+ in DMSO to the blue species by increasing the water percentage in the solution.

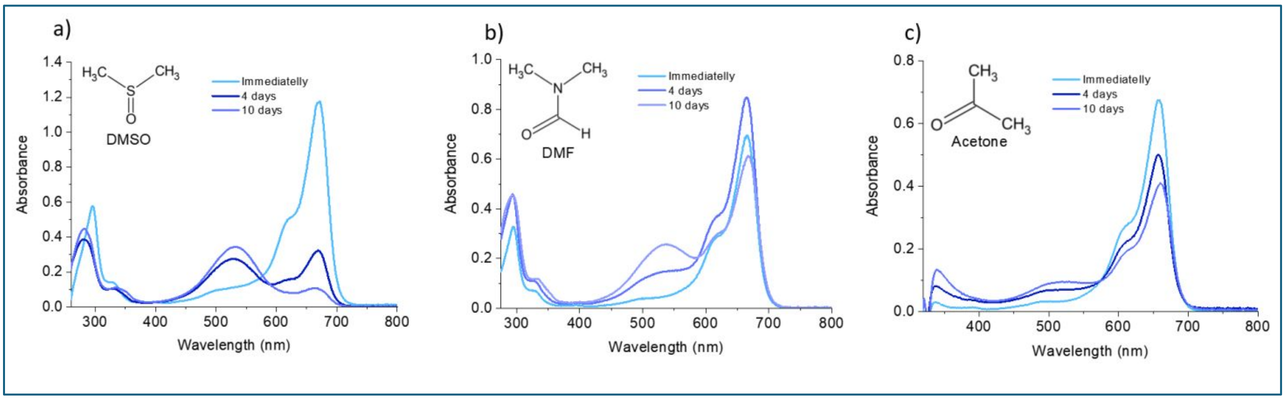

Figure S3. Effect of solvent on the time-dependent formation of MR^+ as indicated in the panels.

EPR results of MB^+-adduct with DMPO

In this setup, DMPO was added to LMB entrapped in Chelex 100 to form an adduct with the free radical produced by one-electron oxidation of LMB. The oxidation of LMB in Chelex was facilitated by UV irradiation (Figure S3). During UV exposure, LMB undergoes oxidation, and in the presence of DMPO, the one-electron-oxidized intermediate is trapped by DMPO. In Figure S3, the spectrum shown by the black line was obtained after irradiating LMB associated with Chelex 100 in the presence of 360 mM DMPO, while the simulated spectrum is displayed with the red line.

The simulated spectrum, based on averaged EPR parameters, is consistent with the expected spectrum of free-rotating MB-DMPO in aqueous solution and is shown in Figure S1b. As previously reported, this simulation did

not match the experimental spectrum corresponding to an immobilized DMPO adduct. The values obtained from the simulation align with those expected for an MB radical centered on sulfur.

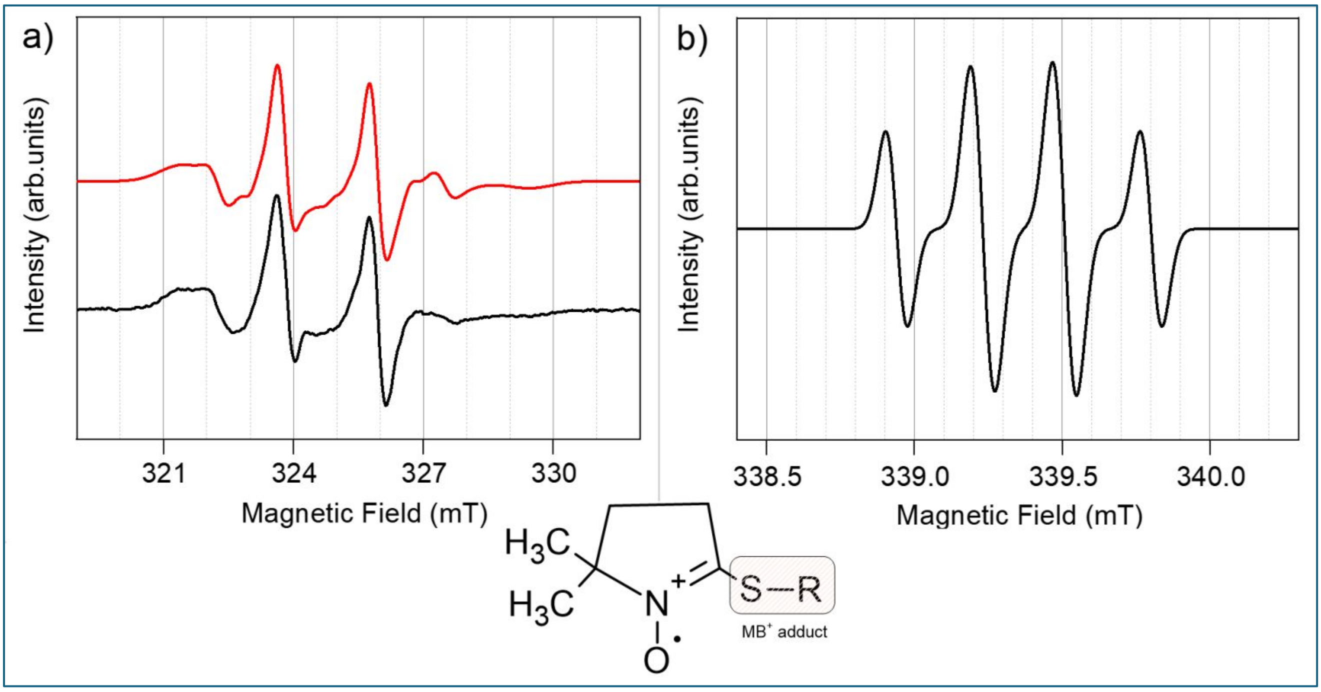

Figure S4. EPR spectra of MB^+-DMPO adduct in Chelex 100 and simulations in the resin and in water. a) Immobilized adduct. The simulation was performed using the EasySpin program in a solid state. Simulation parameters: Microwave frequency 9.1295 GHz, [g_x, g_y, g_z] = [2.0079, 2.0077, 2.0044], g_0 = 2.0067, [A_x^N, A_y^N, A_z^N = [7.84, 15.85, 32.00] Gauss, a_o^N = 18.56 Gauss, [A_x^H, A_y^H, A_z^H = [21.57, 21.27, 19.06] Gauss, a_o^H = 20.63 Gauss, Lw = [2.82, 3.25, 11.3] Gauss, Lw_0 = 5.79 Gauss. b) DMPO Methylene blue anion radical adduct in H_2O solvent. EPR spectrum simulated at room temperature using the EasySpin program with the average EPR parameters from the solid state spectrum. Simulation parameters: Microwave frequency 9.1295 GHz, g_0 = 2.0067, a_o^N = 18.56 Gauss, a_o^H = 20.63 Gauss, and Lw_0 = 5.79 Gauss.

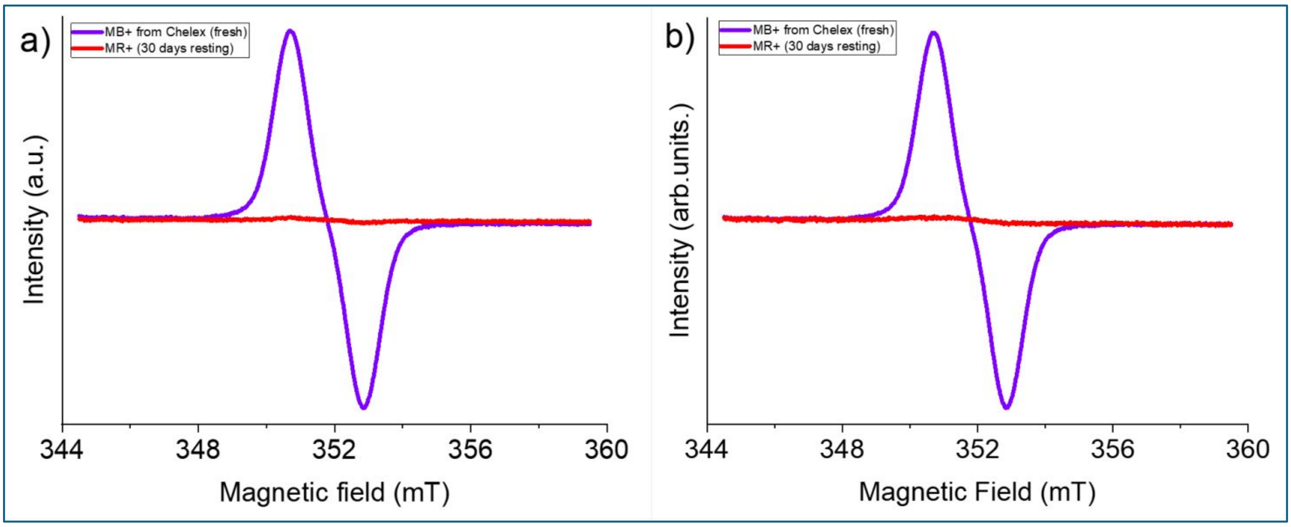

Figure S5. Decay of the EPR signal associated with 30 days of storage of the LMB extracted with a) DMSO and b) Toluene.

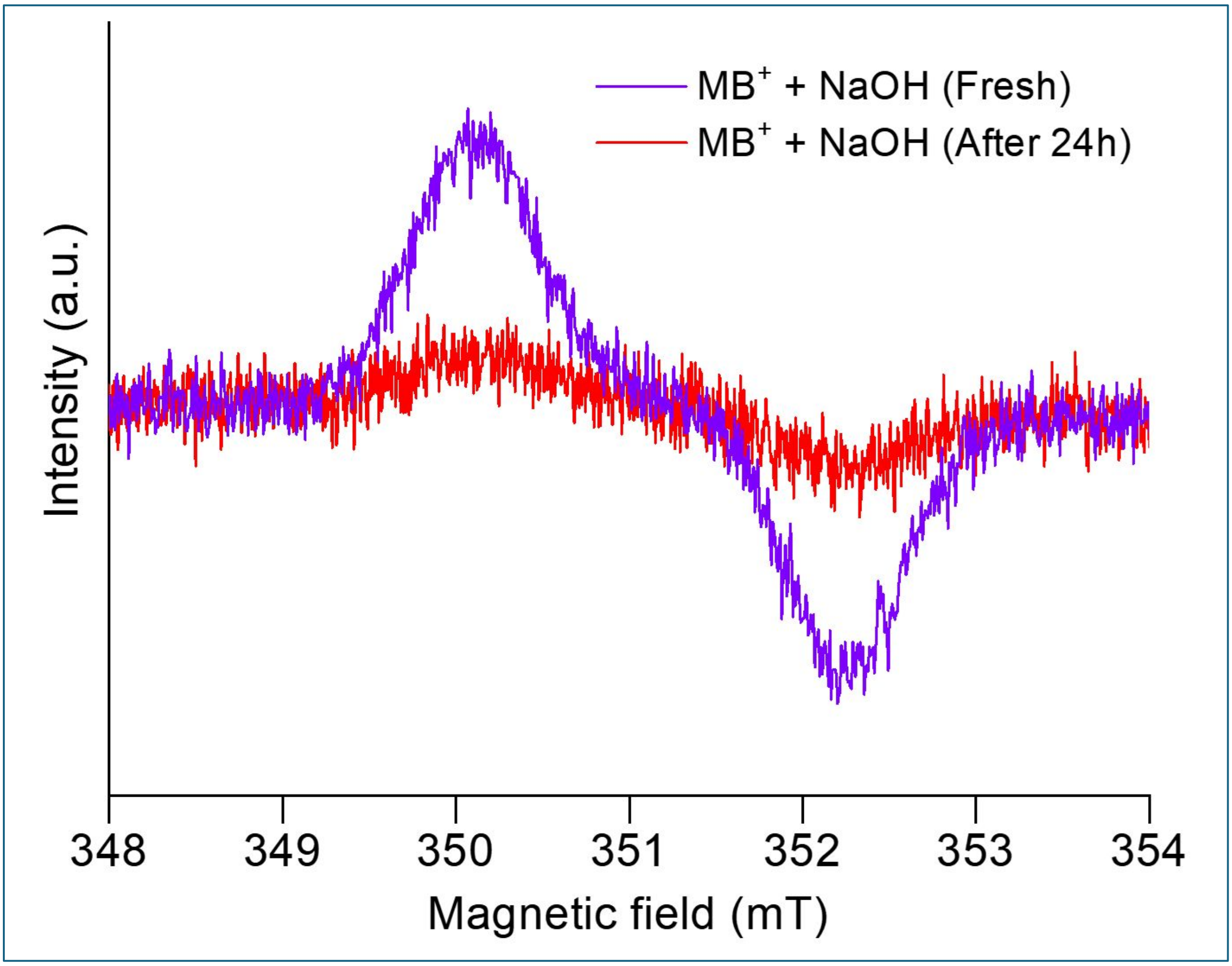

Figure S6. EPR spectra of fresh MB+ OH^- solution (violet line) and after 24 h of resting (red line).

Table S1. Physical properties of the solvents*

Physical Properties	Acetone	DMF	DMSO	Toluene
Dipole moment (D)	20.7	36.7–38.25	46.7	0.31
Polarity index	2.85	3.86	3.9–4.1	2.38
Dielectric constant (ε)	5.1	6.4	7.2	2.4

*According to the reference **[3]**

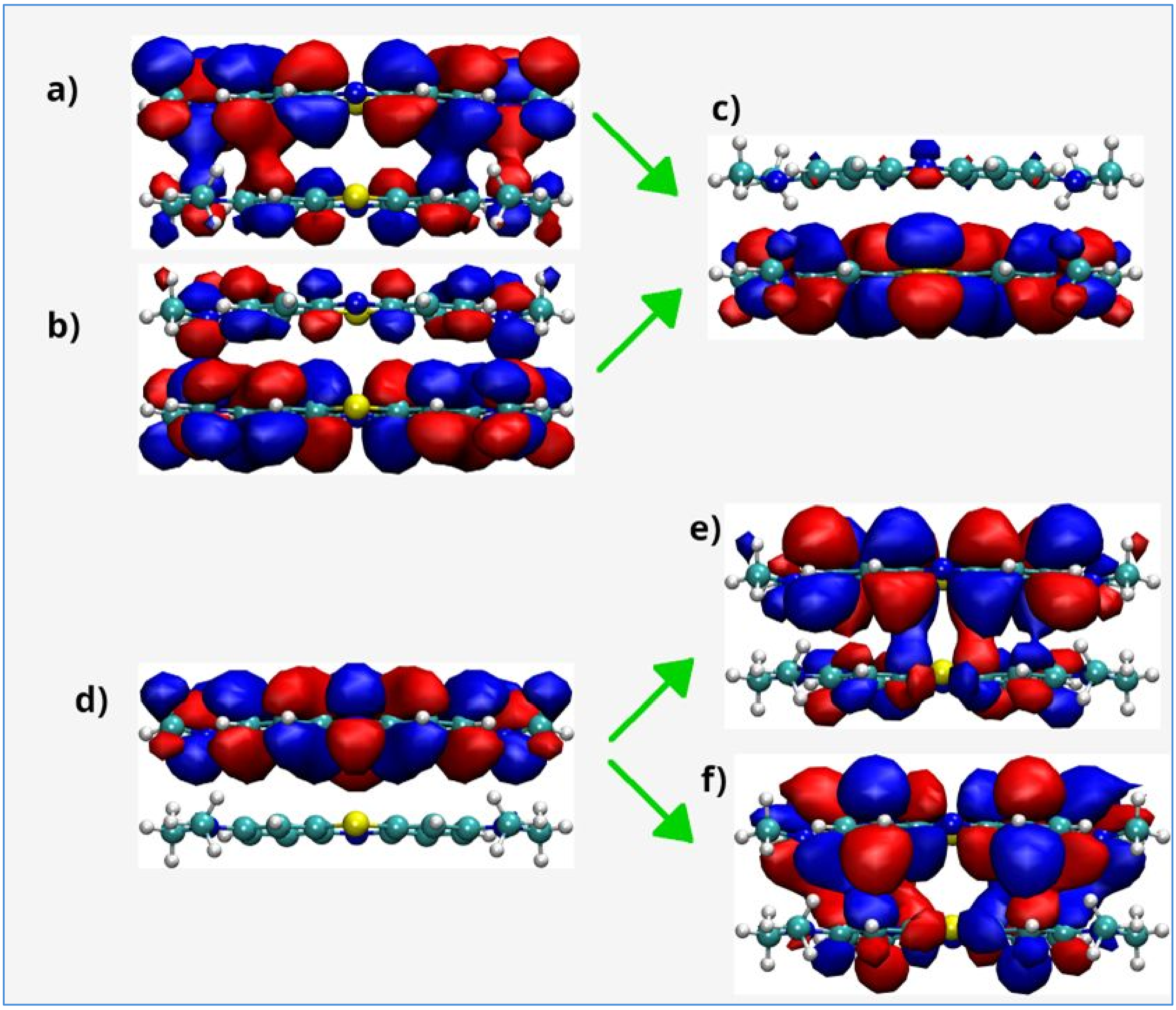

Figure S7. Spin-up states are involved in the strongest optical transition of reduced MB. State a) is HOMO-1 and b) is HOMO transferring charge to c) LUMO+1. Semi-filled state d) SOMO transfers one electron to both states LUMO+4 and LUMO+7.

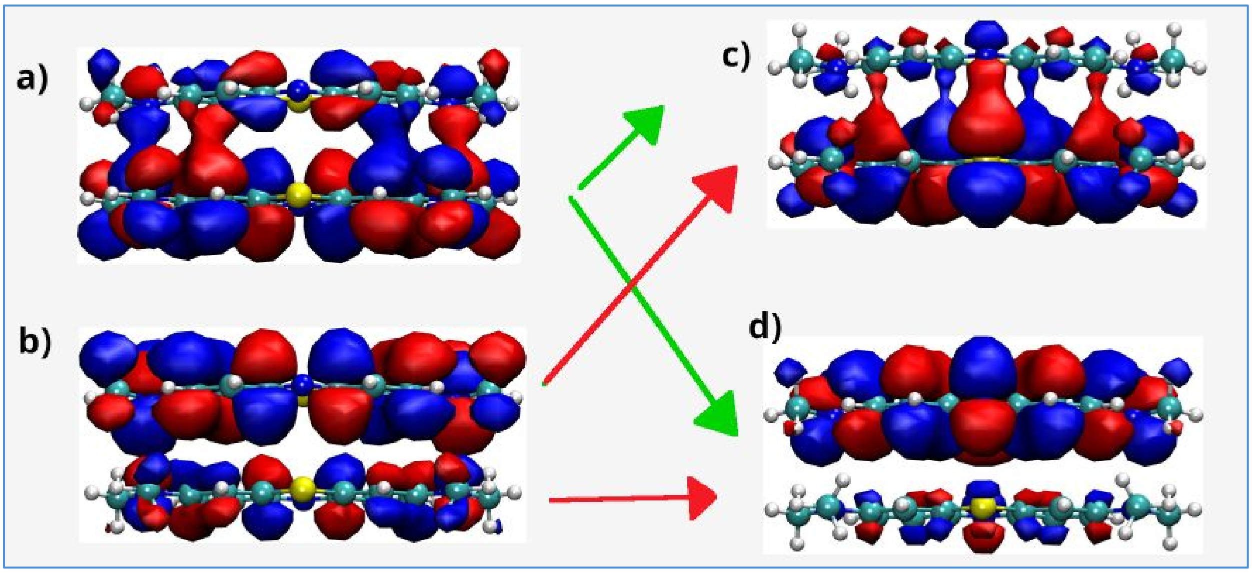

Figure S8. Spin-down states are involved in the strongest optical transition of reduced MB. State a) is HOMO-1 and b) is HOMO transferring charge to c) LUMO+1 and d) SUMO.

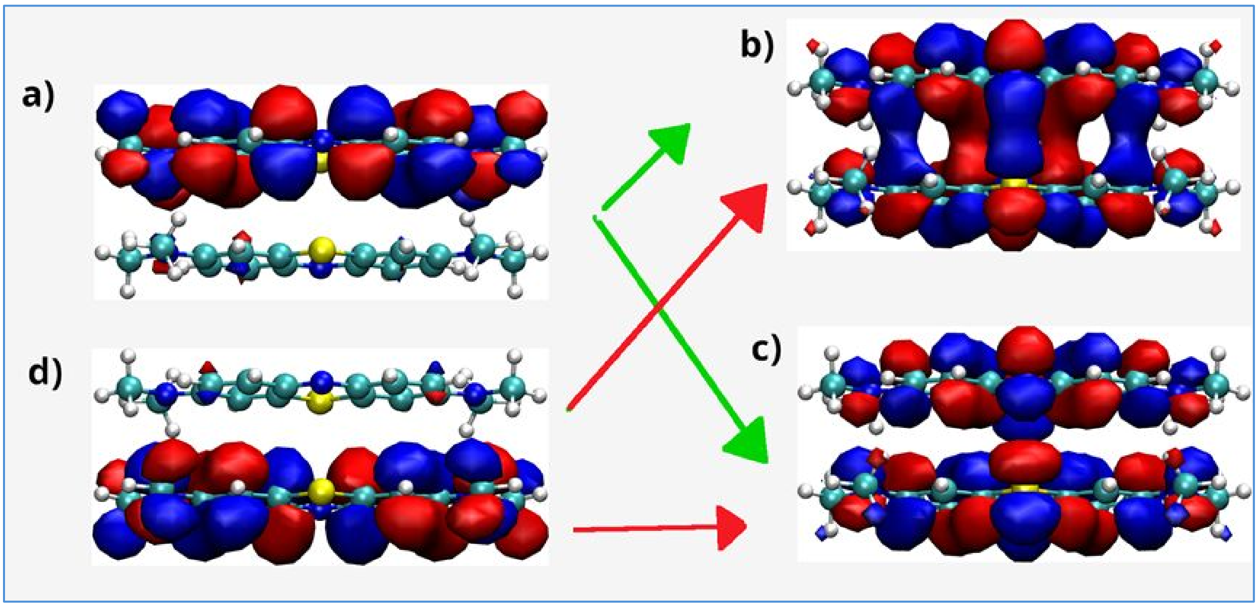

Figure S9. Spin Up states are involved in the strongest optical transition of oxidized MB. State a) is HOMO-2 and d) is SUMO transferring charge to c) LUMO and d) LUMO+1.

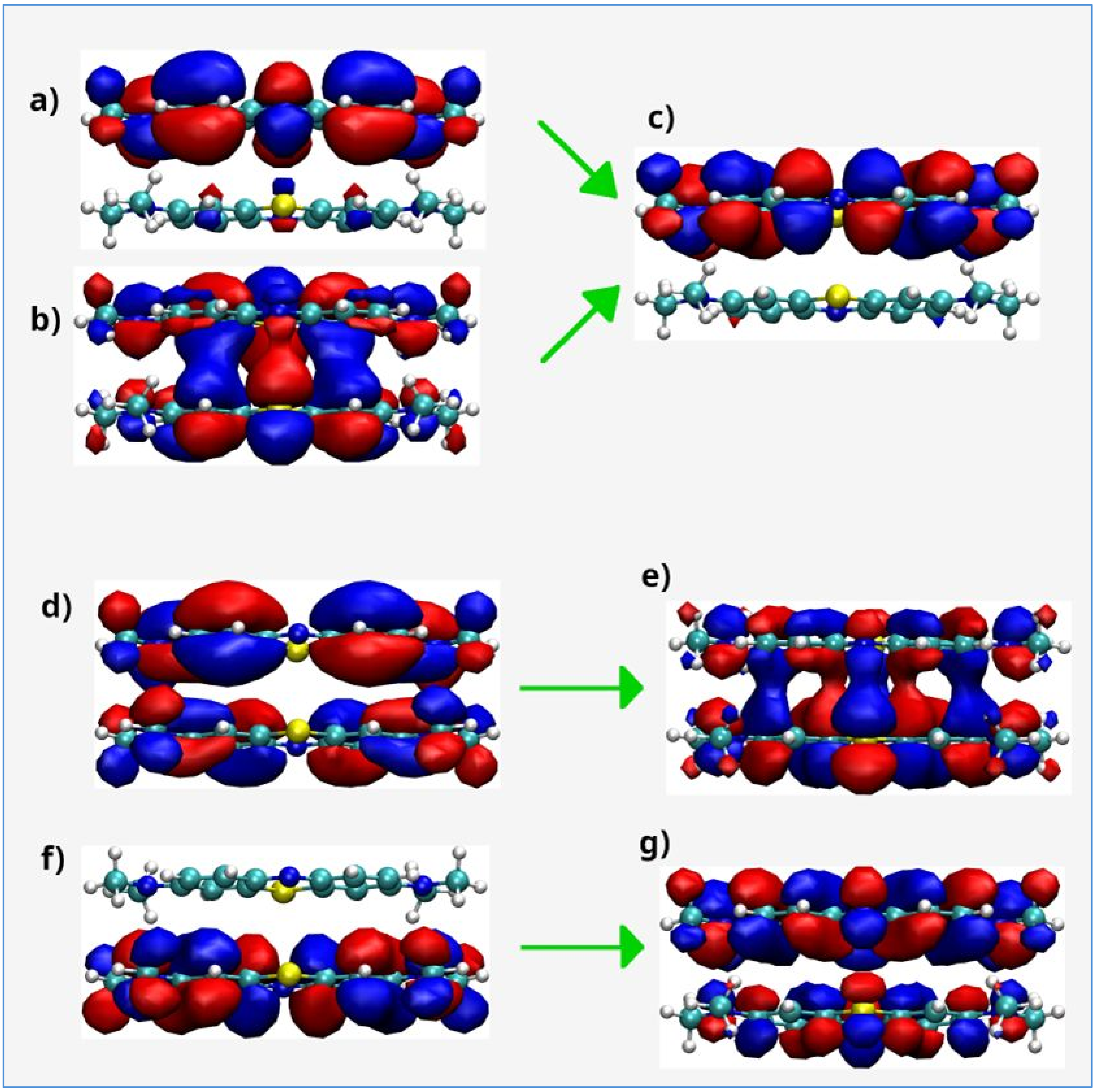

Figure S10. Spin-down states that are involved in the strongest optical transition of oxidized MB. State a) is HOMO-5 and d) is HOMO-3 transferring charge to c) SUMO. d) HOMO-9 transfers an electron to LUMO+1. State HOMO-1 transfers one electron to state LUMO.

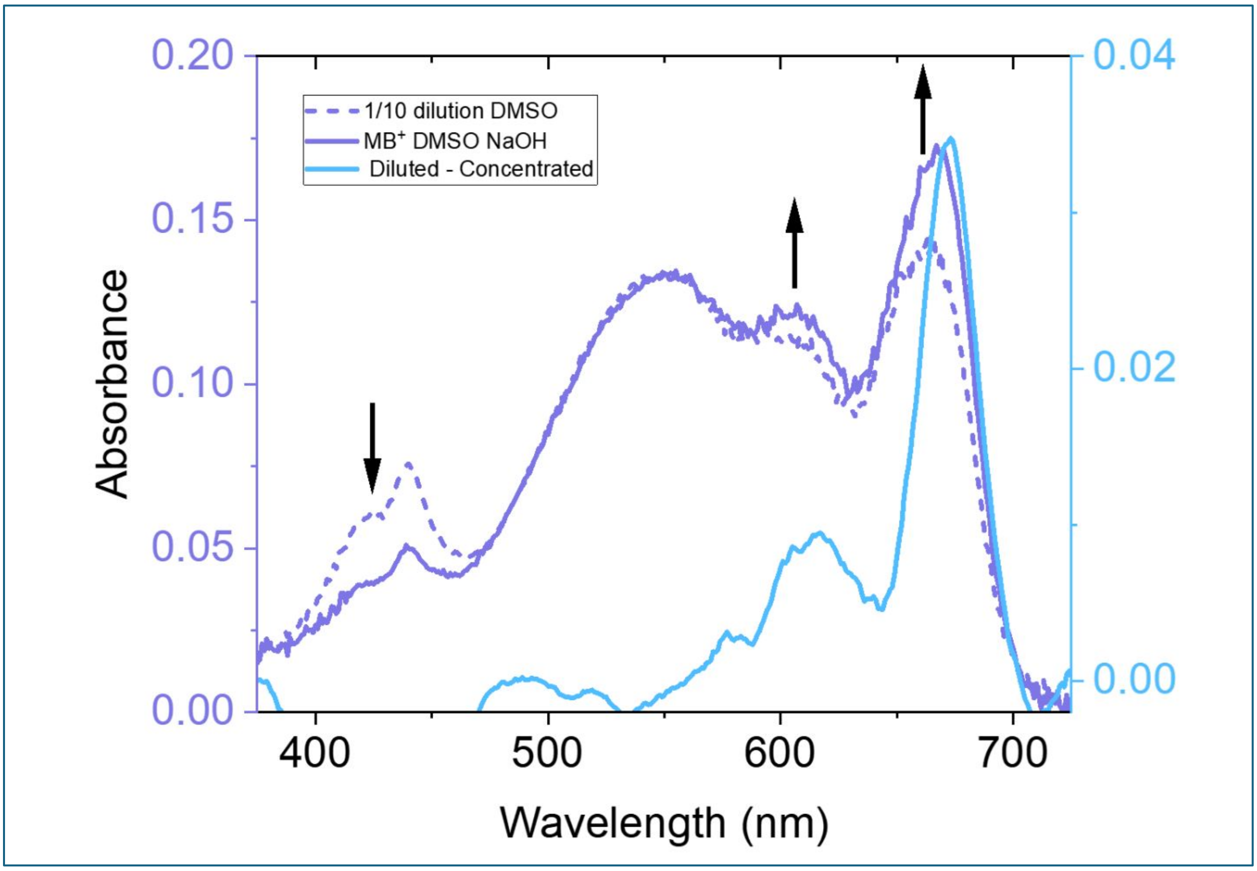

Figure S11. Effect of dilution on the proportion of MB^+ free radicals and aggregates. The spectrum of the diluted sample was corrected according to the optical length used.

Reference

[1] A. Najibi, L. Goerigk, The Nonlocal Kernel in van der Waals Density Functionals as an Additive Correction: An Extensive Analysis with Special Emphasis on the B97M-V and ωB97M-V Approaches, J Chem Theory Comput 14 (2018) 5725–5738. https://doi.org/10.1021/ACS.JCTC.8B00842.

[2] F. Neese, The ORCA program system, Wiley Interdiscip Rev Comput Mol Sci 2 (2012) 73–78. https://doi.org/10.1002/WCMS.81;WEBSITE:WEBSITE:WIRES;WGROUP:STRING:PUBLICATION.

[3] Solvent Physical Properties, (n.d.). https://people.chem.umass.edu/xray/solvent.html (accessed January 11, 2026).
